# Exploring Benzo[h]chromene Derivatives as Agents against Protozoal and Mycobacterial Infections

**DOI:** 10.3390/ph17101375

**Published:** 2024-10-16

**Authors:** Mariano Walter Pertino, Alexander F. de la Torre, Guillermo Schmeda-Hirschmann, Celeste Vega Gómez, Miriam Rolón, Cathia Coronel, Antonieta Rojas de Arias, Carmen A. Molina-Torres, Lucio Vera-Cabrera, Ezequiel Viveros-Valdez

**Affiliations:** 1 Instituto de Química de Recursos Naturales, Universidad de Talca, Campus Lircay, Talca 3480094, Chile; schmeda@utalca.cl; 2 Department of Medical Physics, School of Medicine and Public Health, University of Wisconsin-Madison, Madison, WI 53705, USA; fernandezdel@wisc.edu; 3 Centro para el Desarrollo de la Investigación Científica (CEDIC), Manduvirá 635, Asunción CP 1255, Paraguay; mcvegagomez@gmail.com (C.V.G.); rolonmiriam@gmail.com (M.R.); cathiacoronel@gmail.com (C.C.); rojasdearias@gmail.com (A.R.d.A.); 4 Servicios de Dermatología, Hospital Universitario “José E. González”, Universidad Autónoma de Nuevo León, Monterrey 64460, NL, Mexico; carmen.molinatr@uanl.edu.mx (C.A.M.-T.); lucio.veracb@uanl.edu.mx (L.V.-C.); 5 Facultad de Ciencias Biológicas, Universidad Autónoma de Nuevo León, San Nicolás de los Garza 66455, NL, Mexico; jose.viverosvld@uanl.edu.mx

**Keywords:** benzo[h]chromene, antiprotozoal activity, mycobacterial infections, click chemistry

## Abstract

**Background/Objectives:** In this study, the efficacy of benzo[h]chromene derivatives as antiprotozoal and antimycobacterial agents was explored. **Methods:** A total of twenty compounds, including benzo[h]chromene alkyl diesters and benzo[h]chromene-triazole derivatives, were synthesized and tested against *Trypanosoma cruzi*, *Leishmania braziliensis*, *L. infantum*, and strains of *Mycobacterium abscessus* and *Mycobacterium intracellulare* LIID-01. Notably, compounds **1a**, **1b**, **2a**, and **3f** exhibited superior activity against *Trypanosoma cruzi*, with IC_50_ values of 19.2, 37.3, 68.7, and 24.7 µM, respectively, outperforming the reference drug benznidazole (IC_50_: 54.7 µM). **Results:** Compounds **1b** and **3f** showed excellent selectivity indices against *Leishmania braziliensis*, with SI values of 19 and 18, respectively, suggesting they could be potential alternatives to the commonly used, but more selective, miltefosine (IC_50_: 64.0 µM, SI: 43.0). Additionally, compounds **1a**, **1b**, and **3f** were most effective against *Leishmania infantum*, with IC_50_ values of 24.9, 30.5, and 46.6 µM, respectively. Compounds **3f** and **3h** were particularly potent against various *Mycobacterium abscessus* strains, highlighting their significance given the inherent resistance of these bacteria to standard antimicrobials. **Conclusions:** The sensitivity of *Mycobacterium intracellulare* LIID-01 to these compounds also underscored their potential in managing infections by the Mycobacterium avium–intracellulare complex.

## 1. Introduction

Two significant parasitic diseases in developing countries are Leishmaniasis and Chagas disease (CD), caused by protozoa of the order Kinetoplastidae, *Leishmania* spp. and *Trypanosoma cruzi*, respectively. These are in the most important category of neglected tropical diseases. Chaga’s disease has a four-stage life cycle which has three clinical stages: acute (often asymptomatic, 4–8 weeks), indeterminate (10–20 years), and chronic. In the chronic state when the parasites infiltrate the inner organs (myocardium and intestine), the recurrent process of the disease will continue for years, causing permanent harm [1]. Chagas disease is present in 21 countries in Latin America [2,3,4,5] and in at least ten non-endemic countries, including the United States, Spain, Italy, Japan, and Australia, considering the globalization by immigration [6]. Leishmaniasis is a disease caused by at least 20 species of Leishmania. In humans, the parasite causes both systemic and cutaneous disease, along with many indirect effects [1]. Leishmaniasis is endemic in 98 countries and territories in the world, affecting millions of people. In our research, we have specifically focused on in vitro evaluation of the synthesized compounds on *L. braziliensis* and *L. infantum*. In tropical and subtropical Latin America, *L. braziliensis* is widespread and causes both cutaneous and mucocutaneous diseases. *Leishmania infantum* is a major human disease, with dogs as the confirmed primary infection reservoir. It can contribute to visceral leishmaniasis and lead to death without treatment by infecting vital organs such as the spleen and liver [7]. The extracellular dividing forms of the parasite inside the insect vectors are promastigotes in *Leishmania* spp. and epimastigotes in *T. cruzi*. These models are suitable for preliminary in vitro screening because they are simple to cultivate under laboratory conditions [8,9].

Currently, the only choice for controlling these parasites is chemotherapy. Nevertheless, despite advancements in chemotherapy, there is an urgent and compelling need for the development of new drugs [10]. The drugs available to treat CD until recently were limited to the nitroaromatic heterocycles, nifurtimox, and benznidazole. These compounds are effective in treating only the acute phase of the disease but, in chronically infected adults, showed low efficacy. Moreover, they are associated with numerous side effects such as hypersensitivity, bone marrow depression, peripheral polyneuropathy, and digestive manifestations, among others [11,12]. Similarly, drugs currently used for leishmaniasis therapy have modest effectiveness, require extended treatment, have a high cost, and are accompanied by adverse effects. Furthermore, the parasites have been shown to develop resistance to these drugs [13]. Therefore, the discovery of new drugs is a priority. Only 3.8% of drugs approved by the FDA between 2000 and 2011 were for neglected diseases such as leishmaniasis, CD, and sleeping sickness, which together represent more than 10% of the global burden of diseases [14].

Tuberculosis (TB) is a significant global health concern, primarily caused by *Mycobacterium tuberculosis* (Mtb) and responsible for a substantial number of deaths each year [15]. Although antimycobacterial drugs are available for treatment, only a handful of new chemical entities have progressed to clinical trials [16]. In 2018, the World Health Organization reported around 484,000 new cases of resistance to rifampicin, of which 78% had multidrug-resistant TB (MDR-TB) [17]. In addition to Mtb, there are over 170 different species of mycobacterial nontuberculous (NTM) that cause a broad pathological spectrum such as TB-like pulmonary, extra-pulmonary, and disseminated diseases. Often, NTM does not respond to TB treatments and usually requires long and complex drug therapies, and most NTM are resistant to many antimicrobials [18].

Lapachol, a yellow crystalline pigment found in the bark of the South American lapacho tree (Bignoniaceae), exhibits a wide array of biological activities. The large “lapacho” tree (Hadroanthus impetiginosus (Mart. ex DC.) Mattos; syno-nyms: Tabebuia impetiginosa (Mart. ex DC) Standley and Tabebuia avellanedae Lorentz ex Griseb.) is the botanical source of the prenylated naphthoquinone lapachol. The tree is common in Argentina, Paraguay, Uruguay, and southern Brazil. Lapachol has been used to obtain many bioactive compounds with a variety of structural scaffold; this can be cyclized to form α-lapachone and β-lapachone under strong acidic conditions and to dehydro-α-lapachone and dehydro-β-lapachone with DDQ. However, the synthesis of benzo[h]chromene derivatives from lapachol is not common. Hooker had previously reported the formation of benzo[h]chromene diacetate derivatives, albeit in a mixture of α and β-lapachone in a 1.4:1 ratio [19]. Recently, we have achieved enantioselective synthesis to the β-isomer of benzo[h]chromene diester derivatives [20].

**Figure 1:**
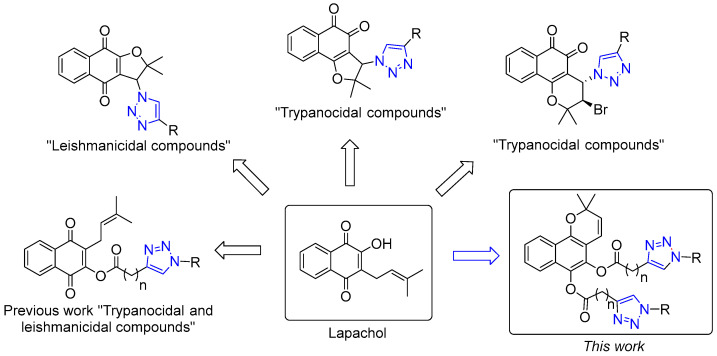
Structural diversity of antiprotozoal lapachol derivatives [21,22,23,24,25,26].

In recent years, several studies have explored the incorporation of triazole rings in quinones like lapachol, β-lapachone, and nor-β-lapachone, producing derivatives with remarkable activity against *T. cruzi* and *Leishmania* (Figure 1) [21,22,23,24,25,26]. Previously, we reported the synthesis, antiprotozoal, and antibacterial activity of new lapachol–triazole derivatives [26].

Following our work on new lead compounds for drug discovery, our present research focuses on the synthesis and evaluation of 18 novel benzo[h]chromene-triazole derivatives. The compounds were assessed against the epimastigote form of *T. cruzi* and promastigotes of *L. braziliensis* and *L. infantum*. Furthermore, we conducted a comprehensive assessment of the compounds’ cytotoxicity on murine fibroblasts and investigated the impact of these compounds on the growth of mycobacteria tuberculous and nontuberculous (NTM).

## 2. Results and Discussion

### 2.1. Chemistry

**Scheme 1:**
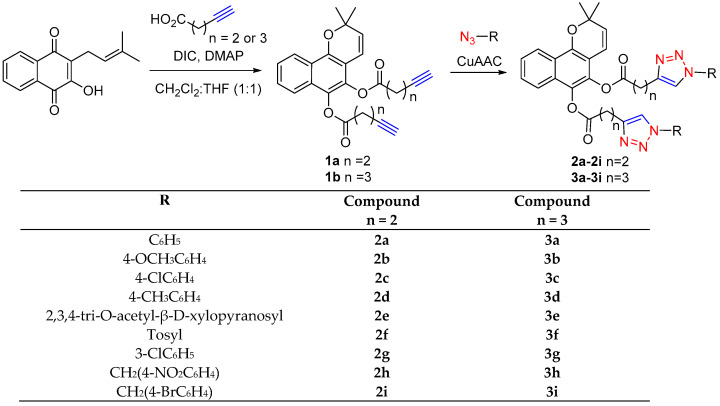
Derivatives of benzo[h]chromene.

The synthesis of the benzo[h]chromene-triazole derivatives (designated as **2a**–**2i** and **3a**–**3i**) followed a stepwise procedure, outlined in Scheme 1. Initially, we generated the benzo[h]chromene diesters, **1a** and **1b**, through the esterification of lapachol. As previously detailed, we achieved the esterification of lapachol by employing various carboxylic acids, N,N-diisopropylcarbodiimide (DIC), and a catalytic amount of 4-dimethylaminopyridine (DMAP) [20]. The synthesis of benzo[h]chromene derivatives involves a two-stage process. First, we produced the monoester of lapachol, which was followed by a concerted pericyclic 6π intra-cyclization. According to the findings, direct esterification in a one-pot reaction is the preferred method for obtaining lapachol monoester, while a stepwise approach is the most suitable for generating benzo[h]chromene derivatives. Two alkynyl esters were prepared and then treated with different aromatic azides using click chemistry to produce 18 new benzo[h]chromene-triazole derivatives. All the products were characterized by spectroscopic means (see the Experimental Section and Supplementary Information).

### 2.2. Biological Evaluation

#### 2.2.1. Antiprotozoal Activity

In this study, we synthesized a range of benzo[h]chromene- diesters (compounds **1a** and **1b**) and benzo[h]chromene-triazole derivatives (compounds **2a**–**2i** and **3a**–**3i**) and investigated their potential as antiprotozoal agents (Table 1 and Table 2). Among the 20 compounds examined, only four exhibited notable antiprotozoal activity against epimastigote forms of *T. cruzi* and promastigotes of *L. braziliensis* and *L. infantum* (Table 1).

Compounds **1a**, **1b**, **2a**, and **3f** showed IC_50_ values of 19.2, 37.3, 68.7, and 24.7 μM, respectively, against *T. cruzi*. These values are comparable to or better than those of the reference compound (benznidazole, IC_50_: 54.7 μM). Additionally, these compounds demonstrated selectivity indexes (SIs) ranging from 3 to 13, while benznidazole had an SI of 14.0.

Remarkably, the alkyl benzo[h]chromene-diester derivatives (compounds **1a** and **1b**) showed better activity against *T. cruzi* than most corresponding benzo[h]chromene-triazole derivatives. Our recent study [26] on lapachol–triazole derivatives demonstrated a similar trend, with lapachol–alkyl esters showing equivalent or superior activity to lapachol–triazole derivatives.

A comparison between alkyl derivatives **1a** and **1b** revealed no significant difference in trypanocidal activity, suggesting that the side chain exerts limited influence on these alkynes. These findings are consistent with the results obtained from previously reported lapachol–alkyl ester derivatives [26]. The derivatives **2f** and **3f** exhibited similar *T. cruzi* activity, with values of 21.4 and 24.7 μM, respectively. Both compounds contain a tosyl group, differing only in chain length. However, compound **2f** was the most cytotoxic among the evaluated derivatives, while compound **3f** showed no cytotoxicity, making it the derivative with the best selectivity index.

The in vitro inhibition of Leishmania proliferation was assessed against two promastigote strains. Compounds **1a**, **1b**, **2f**, and **3f** were the most effective derivatives against *L. braziliensis*, with IC_50_ values of 10.7, 6.7, 14.5, and 17.6 μM, respectively. Compounds **1b** (SI: 19) and **3f** (SI: 18) exhibited the most favorable selectivity indexes. While the reference compound miltefosine displayed greater selectivity, its IC_50_ value was higher than that of the derivatives (IC_50_: 64.0 μM).

The most effective compounds against *L. infantum* were the alkynes **1a** and **1b**, with IC_50_ values of 24.9 and 30.5 µM, respectively. Meanwhile, the best selectivity index was for derivative **3f**, with an SI value of 7, compared with the standard drug miltefosine (SI: 109.7). There is a variation in the activity against *L. infantum*. However, alternative medications are required because miltefosine resistance has been reported in recent papers [27,28].

#### 2.2.2. Antibacterial Activity

The emergence of antibiotic resistance has become a significant global challenge in the context of infectious diseases, particularly those caused by mycobacteria. The pressing need for innovative treatment options has driven extensive research into the evaluation of a diverse array of compounds for their potential efficacy against mycobacterial infections [29]. Among these, natural products and their semi-synthetic derivatives have played a pivotal role, exemplified by the utilization of rifampicin, streptomycin, amikacin, capreomycins, and cycloserine as essential antitubercular agents [30]. Furthermore, the naphthoquinone lapachol has demonstrated potential activity against tuberculosis strains [31,32].

Ester prodrugs offer a classical strategy for masking polar alcohol and carboxylic acid functionalities and improving cell permeability [33]. This property is of paramount importance in the search for novel antituberculosis agents, as mycobacterial cell walls are characterized by complex structures that are highly hydrophobic and resistant to the permeation of many compounds [34]. In this study, we observed that the benzo[h]chromene derivates, can be considered interesting compounds for the discovery of novel antimycobacterial molecules to treat NTM infections. Notably, we have identified derivatives **3f** and **3h** as exhibiting substantial activity against all analyzed *M. abscessus* strains, which is remarkable considering the inherent resistance of *M. abscessus* to most conventionally used antimicrobials. The primary screens for *M. abscessus* often yield hit rates lower than 0.1% [35].

On the other hand, the most susceptible strain was found to be *M. intracellulare* LIID-01. This finding carries significant clinical relevance, as the Mycobacterium avium–intracellular complex is a primary cause of NTM infections in individuals with HIV/AIDS [36]. The limited availability of active drugs has resulted in prolonged and challenging treatment regimens [37]. This underscores the importance of our investigation into benzo[h]chromene as potential therapeutic agents for addressing antibiotic resistance in the context of NTM infections. In a related context, we observed that compound **3f** exhibited notable antimicrobial activity against both Gram-positive and Gram-negative bacteria, as detailed in Table 3. This finding is particularly significant due to the escalating concern surrounding antibiotic resistance within the latter group of microorganisms [38]. Notably, this resistance extends to pathogens such as mycobacteria, where the membrane is recognized as a pivotal factor contributing to this resistance [39]. The research conducted by Sangwan et al. in 2023 [40] has recently provided compelling evidence for the potential of compounds incorporating 1,2,3-triazole hybrids with amine-ester functionality.

### 2.3. ADME In Silico Approach

We also studied the ADME properties of the compounds predicted using SwissADME server (Table 4). The Lipinski’s Rule, also known as the “Rule of Five”, is a fundamental concept in drug discovery and development. It was introduced by Lipinski et al. [41] to delineate the physicochemical parameters associated with oral drug absorption and the likelihood of becoming successful oral drugs. According to this rule, compounds are more likely to be orally active if they have certain physicochemical properties, such as a molecular weight below 500, a Log P (octanol–water partition coefficient) less than 5, no more than 5 hydrogen bond donors, and no more than 10 hydrogen bond acceptors. Only compounds **1a** and **1b** comply with these rules, with no violations observed for compound **1a** and one violation for compound **1b**. The remaining compounds do not adhere to these criteria, with two violations identified for compounds **2e**, **3e**, **2h**, and **3h**, and three violations for the remaining compounds. While Lipinski’s Rule serves as a guideline for drug design, there are exceptions where molecules outside these parameters are still used as drugs, highlighting the complexity of drug development and the importance of considering multiple factors in drug design and optimization [42].

The topological polar surface area (TPSA) is another feature of interest, as drugs with a low TPSA (≤140 Å^2^) typically have higher oral bioavailability [43]. Among the twenty synthesized compounds, twelve demonstrate favorable TPSA values (refer to Table 4). Predictions suggest limited gastrointestinal absorption for the compounds, except for compounds **1a** and **1b**, which may be attributed to their low calculated aqueous solubility (LogS). Additionally, only compound **1a** exhibits the capability to traverse the blood–brain barrier (BBB). Considering both experimental bioactivity and in silico data, compound **1a** emerges with a favorable drug-likeness profile and excellent chemical stability, positioning it as the leading candidate.

**Table 4:** Physicochemical and pharmacokinetic descriptors calculated with SwissADME.

	Physicochemical Properties					Lipophilicity	Water Solubility	Pharmacokinetics		
	MW ^1^	RB ^2^	HBA ^3^	HBD ^4^	TPSA ^5^	Log Po/w ^6^	(mol/L)	GI abs ^7^	BBB ^8^	log Kp ^9^
**1a**	402.44	8	5	0	61.83	4.73	9.14 × 10^−4^	High	Yes	−5.47
**1b**	430.49	10	5	0	61.83	5.23	1.79 × 10^−4^	High	No	−5.14
**2a**	640.69	12	9	0	123.25	5.39	1.35 × 10^−6^	Low	No	−5.74
**3a**	668.74	14	9	0	123.25	6.16	2.52 × 10^−7^	Low	No	−5.40
**2b**	700.74	14	11	0	141.71	5.51	6.97 × 10^−7^	Low	No	−6.14
**3b**	728.79	16	11	0	141.71	5.87	1.30 × 10^−7^	Low	No	−5.80
**2c**	709.58	12	9	0	123.25	6.48	7.35 × 10^−8^	Low	No	−5.26
**3c**	737.63	14	9	0	123.25	7.10	1.40 × 10^−8^	Low	No	−4.93
**2d**	668.74	12	9	0	123.28	5.97	2.49 × 10^−7^	Low	No	−5.39
**3d**	696.79	14	9	0	123.25	6.64	4.70 × 10^−8^	Low	No	−5.06
**2e**	1004.94	24	23	0	299.51	2.92	9.60 × 10^−6^	Low	No	−10.94
**3e**	1033.00	26	23	0	299.51	3.18	1.77 × 10^−6^	Low	No	−10.60
**2f**	796.87	14	13	0	208.29	5.66	3.75 × 10^−8^	Low	No	−6.87
**3f**	840.96	16	13	0	208.29	5.50	1.53 × 10^−9^	Low	No	−6.08
**2g**	709.58	12	9	0	123.25	6.60	7.35 × 10^−8^	Low	No	−5.26
**3g**	737.63	14	9	0	123.25	7.19	1.40 × 10^−8^	Low	No	−4.93
**2h**	758.74	16	13	0	214.89	4.49	5.84 × 10^−8^	Low	No	−6.79
**3h**	786.79	18	13	0	214.89	5.03	1.08 × 10^−8^	Low	No	−6.45
**2i**	826.53	14	9	0	123.25	6.67	8.57 × 10^−8^	Low	No	−5.97
**3i**	854.59	16	9	0	123.25	7.47	1.62 ×10^−8^	Low	No	−5.64

^1^ Molecular weight (g/mol); ^2^ number of rotatable bonds; ^3^ number of hydrogen bond acceptors; ^4^ number of hydrogen bond donors; ^5^ topological polar surface area [44]; ^6^ average of iLOGP, XLOGP, WLOGP, MLOGP, and SILICOS-IT predictions (consensus Log Po/w) [45]; ^7^ gastrointestinal absorption; ^8^ blood–brain barrier permeation; ^9^ skin permeation: QSPR model (cm/s) [46].

## 3. Materials and Methods

### 3.1. Chemistry

#### 3.1.1. General Overview

^1^H NMR and ^13^C NMR spectra were recorded at 400 MHz for ^1^H and 100 MHz for ^13^C, respectively using a Bruker Avance 400 instrument (Bruker Corporation, Rheinstetten, Baden-Württemberg, Germany). Chemical shifts (δ) are reported in parts per million relative to the residual solvent signals, and coupling constants (*J*) are reported in Hertz. High-resolution ESI mass spectra were obtained from a Fourier transform ion cyclotron resonance (FT-ICR) mass spectrometer, an RF-only hexapole ion guide, and an external electrospray ion source (Bruker Daltonics Inc., Billerica, MA, USA). Flash column chromatography was carried out using silica gel 60 (230–400 mesh) from Merck KGaA, Darmstadt, Hesse, Germany. Analytical thin layer chromatography (TLC) was performed using silica gel on aluminum sheets (Merck KGaA, Darmstadt, Hesse, Germany).

#### 3.1.2. General Procedure Followed for Esterification

A solution of the acid (2.0 equiv.) in a mixture of DCM (0.5 mL) with DIC (2.0 equiv.) was stirred for 10 min at room temperature. Then, lapachol with a catalytic amount of DMAP dissolved in DCM (0.5 mL) were added together, and the reaction was stirred for 12 h at room temperature. Next, 1 mL of THF followed by the correspondent azide (2.2 equiv.) was added, and the reaction mixture was treated with solutions of CuSO_4_·5H_2_O (20 mol%) and sodium ascorbate (40 mol%), enabling the formation of Cu(I) in situ. After the reaction completion (monitored by TLC), the reaction mixture was diluted with 20 mL of DCM and washed successively with NaHCO_3_ (sat.), brine, and water. The extract was dried over Na_2_SO_4_ and purified by silica gel column chromatography.

### 3.2. Biology

#### 3.2.1. Antiprotozoal Assays

For in vitro studies of *T. cruzi*. the clone CL-B5 was used. Parasites were stably transfected with the *Escherichia coli* β-galactosidase gene (lacZ), provided by Facultad de Farmacia (Universidad Complutense de Madrid). Epimastigotes were grown at 28 °C in liver infusion tryptose broth (Difco, Detroit, MI, USA) with 10% fetal bovine serum (FBS) (Gibco, Carlsbad, CA, USA), penicillin (Ern S.A., Barcelona, Spain), and streptomycin (Reig Jofré S.A., Barcelona. Spain), as described previously [8], and harvested during the exponential growth phase. Briefly, the screening assay was performed in 96-well microplates (Sarstedt, Inc., Newton, NC, USA) with cultures that had not reached the stationary phase. Briefly, epimastigotes were seeded at 2 × 10^5^ parasites/mL in 200 µL growth media. The plates were then incubated with the respective compounds at 28 °C for 72 h. Afterwards, 50 µL of CPRG solution was added, resulting in a final concentration of 200 µM. The plates were incubated at 37 °C for an additional 4 h and then read at 595 nm. Each concentration was tested in triplicate [9]. The efficacy of each compound was estimated by calculating the IC_50_ values. These values were calculated from the sigmoidal dose–response curve adjustment using the statistical software program Graph-Pad Prims 3.0. Benznidazole was used as a reference drug.

The culture of *L. braziliensis* (MHOM/CO/88/UA301) and *L. infantum* (MCAN/ES/92/BCN83) was obtained from the Facultad de Farmacia, Universidad Complutense de Madrid, Spain.

The maintenance of the strains, the form of cultivation, and the isolation of shape promastigota followed the procedures described by Roldós et al. [8]. The promastigotes were grown at 22 °C in Schneider’s Drosophila medium supplemented with 20% FBS. The assay was performed using a modification of a previous method [47]. Promastigotes (2 × 10^6^ parasites/well) were cultured in 96-well plastic plates. Compounds were dissolved in dimethylsulfoxide (DMSO). Different dilutions of the compounds up to a 200 µL final volume were added. After 48 h at 26 °C, 20 µL of 2 mM resazurin solution was added and the oxidation–reduction was quantified at 570 and 600 nm. The solution of resazurin was prepared at 2.5 mM in phosphate-buffered solution (PBS), pH 7.4, and filtered through 0.22 µM prior to use. All tests were carried out in triplicate. Resazurin sodium salt was obtained from Sigma–Aldrich (St. Louis, MO, USA) and stored at 4 °C protected from light. The efficacy of each compound was estimated by calculating the IC_50_ values.

#### 3.2.2. Cytotoxicity

The cell line used was L929, which was grown in Minimal Essential Medium (SIGMA) supplemented with 10% heat-inactivated FBS, penicillin G (100 U/mL), and streptomycin (100 µg/mL). The cell cultures were maintained at 37 °C in a humidified 5% CO_2_ atmosphere. The procedure for cell viability measurement was evaluated with resazurin by a colorimetric method. The cells were plated in 96-microtitre plates at 3 × 10^4^ cells per well in 100 µL growth medium. The cells were grown overnight at 37 °C with 5% CO_2_. Thereafter, the medium was removed and the compounds were added in 200 µL medium for 24 h. After incubation, 20 µL of 2 mM resazurin solution was added to each well. The plates were incubated for 3 h to allow optimal oxidation–reduction. The reduction of resazurin was determined by dual wavelength absorbance measurement at 490 and 595 nm. The background was subtracted. Each concentration was assayed three times. Medium and drug controls were used in each test as blanks [48].

#### 3.2.3. Bacterial Strains and Culture Conditions

Six clinical isolates belonging to NTM, including three strains of *M. abscessus*, two of *M. intracellulare*, and one of *M. fortuitum*, as well as two strains of *M. tuberculosis* were obtained from the Laboratorio Interdisciplinario y de Investigación Dermatológica (LIID) belonging to the Hospital Universitario José E. González (Monterrey, Nuevo León, México). The reference isolate *M. tuberculosis* H37Rv ATCC 27294 was obtained from the American Type Culture Collection (ATCC). The strains were activated from the frozen stocks in Lowenstein–Jensen medium and blood agar and incubated at 37 °C for 7–14 days. Gram-negative—*Escherichia coli*, *Pseudomonas aeruginosa*, and *Salmonella typhi*—and Gram-positive—*Staphylococcus aureus*, *Listeria innocua*, *Listeria monocytogenes*, *Enterococcus faecalis*, *Bacillus cereus*, and *Micrococcus luteus—*bacteria were subcultured on Brain Heart Infusion Broth agar and incubated at 35 °C for 24 h prior to the assays. All the strains were identified to the species level by a biochemical test or by PCR [49].

#### 3.2.4. Determination of the Minimum Inhibitory Concentration (MIC)

MIC, defined as the minimal concentration of a compound which prevents visible growth of the bacteria, was determined by measurement of turbidity in the wells using the broth microdilution method (CLSI, 2011). Subject to NTM strains, cation-adjusted Mueller–Hinton broth (CA-MHB) was used for bacteria and rapidly growing mycobacteria (*M. abscessus* and *M. fortuitum*) and Middlebrook 7H9 broth supplemented with oleic albumin dextrose catalase (OADC) for slow growing mycobacteria (*M. intracellulare*). The MIC was determined after 24 h at 37 °C for Gram-positive and -negative bacteria, 72 h at 37 °C for *M. abscessus* and *M. fortuitum* strains, and after 7 to 10 days for *M. intracellulare* strains. The compounds were evaluated from 3.12 to 200 μg/mL. The experiments were performed in duplicates.

### 3.3. ADME In Silico Approach

The SwissADME server (http://www.swissadme.ch/) was used to analyze the compounds’ physicochemical characteristics, ADME (absorption, distribution, metabolism, and excretion), pharmacokinetic characteristics, drug-like nature, and biocompatibility [45]. In summary, each compound was projected to have 42 descriptors, which included physicochemical characteristics, lipophilicity, water solubility, and pharmacokinetics. The acceptability of the compounds based on bioavailability score (drug-likeness) may be evaluated using the descriptors that were derived [45].

## 4. Conclusions

In conclusion, our research provides valuable insights into the potential of benzo[h]chromene derivatives as antiprotozoal and antimycobacterial agents. Among the synthesized compounds, four showed promising antiprotozoal activity against *T. cruzi* and *Leishmania* strains, with compounds **1a**, **1b**, **2a**, and **3f** exhibiting particularly noteworthy results, surpassing the reference compound benznidazole in some cases. The alkyl benzo[h]chromene-ester derivatives (**1a** and **1b**) demonstrated equal efficacy, suggesting that the side chain’s length had minimal influence on their activity, a finding consistent with previous research.

Additionally, these compounds exhibited potential against *Leishmania* strains, with **1b** and **3f** demonstrating the best selectivity indices. Although miltefosine displayed higher selectivity, it had a higher IC_50_ value, making the newly synthesized derivatives promising alternatives, especially considering reported miltefosine resistance.

Furthermore, the study explored the potential of benzo[h]chromene derivatives in combating *mycobacterial nontuberculous* (NTM) infections, a significant challenge in the field of antimicrobial therapy. Compounds **3f** and **3h** demonstrated effectiveness against various *M. abscessus* strains, highlighting their potential as antimycobacterial agents. Notably, *M. intracellulare* LIID-01, which is a critical concern for HIV/AIDS patients, exhibited sensitivity to these compounds. Given the limited options for treating NTM infections, the findings suggest that benzo[h]chromene derivatives may hold promise for developing novel antimycobacterial molecules.

In summary, this research opens new avenues for the development of compounds with therapeutic potential against protozoal and mycobacterial infections. These findings emphasize the importance of exploring novel chemical entities as potential treatments for challenging infectious diseases.

## Figures and Tables

**Table 1:** Antiprotozoal activity of the lapachol–triazole derivatives on *Leishmania brazilensis* (*L. b*) and *Leishmania infantum* (*L. i*) promastigotes, and *T. cruzi* epimastigotes and cytotoxicity on NCTC929 fibroblast cells.

Compound	Leishmanicidal Activity (µM)		Trypanocidal Activity (µM)	Cytotoxicity (µM)	Selectivity Index *		
	*IC_50_—L. b*	*IC_50_—L. i*	*IC_50_ T. cruzi*	*CC_50_—L929*	L929/ *L. b*	L929/ *L. i*	L929/ *T. cruzi*
**1a**	10.7	24.9	19.2	106.4	10	4	6
**1b**	6.7	30.5	37.3	128	19	4	3
**2a**	>256	>256	68.7	>256	NE	NE	>4
**3a**	>256	>256	>256	>256	NE	NE	NE
**2b**	>256	>256	204.3	>256	NE	NE	>1
**3b**	>256	>256	>256	>256	NE	NE	NE
**2c**	>256	>256	>256	>256	NE	NE	NE
**3c**	>256	>256	>256	>256	NE	NE	NE
**2d**	>256	>256	>256	>256	NE	NE	NE
**3d**	>256	>256	>256	>256	NE	NE	NE
**2e**	>256	>256	>256	>256	NE	NE	NE
**3e**	>256	>256	>256	>256	NE	NE	NE
**2f**	14.5	38.2	21.1	31.8	2	1	2
**3f**	17.6	46.6	24.7	312.2	18	7	13
**2g**	>256	>256	>256	>384	>2	>2	>2
**3g**	>256	>256	146.5	>384	>2	>2	>3
**2h**	>256	>256	>256	>256	NE	NE	NE
**3h**	>256	>256	>256	>256	NE	NE	NE
**2i**	>256	>256	>256	>256	NE	NE	NE
**3i**	>256	>256	>256	>256	NE	NE	NE
Miltefosine	64.0	25.1	NA	2754	43.0	109.7	NA
Benznidazole	NA	NA	54.7	769	NA	NA	14.0

* SI = (*CC_50_ L929*/*IC_50_* parasites); NA: not applicable; NE: not estimable; *IC_50_* and *CC_50_* values > 256 µM.

**Table 2:** MIC values (µg/mL) of samples against mycobacteria clinical strains. Only compounds active at <200 μg/mL in some mycobacterial strains are presented.

Strain	1a	3e	2f	3f	3h	* Rifampicin	* Linezolid	* Imipenem
*M. tuberculosis* CIPTIR *-*D152	>200	>200	>200	>200	>200	32	NA	NA
*M. tuberculosis* CIPTIR-F296	>200	>200	>200	>200	>200	1	NA	NA
*M. tuberculosis ATCC H37Rv*	>200	>200	>200	>200	>200	2	NA	NA
*M. abscessus* LIID-01	>200	>200	>200	100	100	NA	1	32
*M. abscessus* LIID-02	>200	>200	>200	100	100	NA	1	32
*M. abscessus* LIID-03	>200	>200	>200	100	100	NA	1	8
*M. fortuitum* LIID-01	>200	>200	>200	100	>200	NA	8	4
*M. intracellulare* LIID-01	50	50	50	100	>200	NA	2	1
*M. intracellulare* LIID-02	>200	>200	>200	>200	>200	NA	8	>64

* Reference drug, NA: not applicable.

**Table 3:** MIC values (µg/mL) of samples against selected clinical strains of bacteria. Only compounds active at <200 μg/mL in some mycobacterial strains are presented.

Strain	1a	3e	2f	3f	3h	* Gentamicin
*E. coli* (*G*−)	>200	>200	>200	100	>200	0.2
*P. aeruginosa* (*G*−)	>200	>200	>200	100	>200	0.78
*S. typhi* (*G*−)	>200	100	>200	>200	100	0.1
*S. aureus* (*G*+)	100	>200	>200	100	100	0.1
*L. innocua (G*+)	>200	>200	>200	100	>200	1.56
*L. monocytogenes* (*G*+)	>200	>200	>200	100	>200	0.2
*E. faecalis* (*G*+)	>200	>200	>200	>200	>200	0.78
*B. cereus* (*G*+)	>200	>200	>200	100	>200	0.78
*M. luteus* (*G*+)	>200	>200	>200	100	>200	0.78

* Reference drug.

## Data Availability

The data are contained within the article or Supplementary Materials.

## Supplementary Materials

The following supporting information can be downloaded at https://www.mdpi.com/article/10.3390/ph17101375/s1. Figures S1–S40: ^1^H and ^13^C spectra of the synthesized compounds.
