# Supplementary material for: Exploring Benzo[h]chromene Derivatives as Agents against Protozoal and Mycobacterial Infections
Source: Pharmaceuticals (Basel). 2024 Oct 16;17(10):1375. doi: 10.3390/ph17101375 (PMC11510191; doi:10.3390/ph17101375)
Supplement: Supplementary file 1 [file pharmaceuticals-17-01375-s001.zip › pharmaceuticals-3265124-supplementary.pdf]

Supplementary materials

Exploring Benzo[h]chromene Derivatives as Agents against Protozoal and Mycobacterial Infections

Mariano Walter Pertino [1,*], Alexander F. de la Torre [2], Guillermo Schmeda-Hirschmann [1], Celeste Vega Gómez [3], Miriam Rolón [3], Cathia Coronel [3], Antonieta Rojas de Arias [3], Carmen A. Molina-Torres [4], Lucio Vera-Cabrera [4] and Ezequiel Viveros-Valdez [5]

[1] Instituto de Química de Recursos Naturales, Universidad de Talca, Campus Lircay, Talca 3480094, Chile
[2] Department of Medical Physics, School of Medicine and Public Health, University of Wisconsin-Madison, Madison, WI 53705, USA
[3] Centro para el Desarrollo de la Investigación Científica (CEDIC), Manduvirá 635, CP 1255, Asunción, Paraguay
[4] Servicios de Dermatología, Hospital Universitario "José E. González", Universidad Autónoma de Nuevo León, Monterrey 64460, NL, Mexico
[5] Facultad de Ciencias Biológicas, Universidad Autónoma de Nuevo León, San Nicolás de los Garza 66455, NL, Mexico
* Correspondence: mwalter@utalca.cl

We previously reported the characterization of compounds **3a**-**3g**.

A.F. de la Torre, A. Ali, B. Westermann, G. Schmeda-Hirschmann, M.W. Pertino. An efficient cyclization of lapachol to new benzo[*h*]chromene hybrid compounds: a stepwise vs. one-pot esterification-click (CuAAC) study, New J. Chem. 42 (2018) 19591–19599.

Characterization and spectra of selected compounds

Compound 2a. Lapachol (24.2 mg, 0.1 mmol) and 4-pentynoic acid (20 μL, 0.2 mmol), azidobenzene solution (440 μL, 0.22 mmol, *c* = 0.5 M) were reacted in DCM:THF 1:1 *vv* (2 mL) according to the stepwise esterification-click general procedure. Flash column chromatography purification (*n*-hexane/EtOAc 6:4) afforded **2a** (51 mg, 78%) as a light yellow solid. ^{1}H NMR (400 MHz, $CDCl_3$) δ 7.99 (d, *J* = 8.2 Hz, 1H), 7.74 (d, *J* = 7.7 Hz, 2H), 7.54 (t, *J* = 8.5 Hz, 4H), 7.30 (ddd, *J* = 28.3, 17.9, 6.9 Hz, 10H), 6.06 (d, *J* = 9.9 Hz, 1H), 5.43 (d, *J* = 9.9 Hz, 1H), 3.04 (m, 4H), 2.97 (t, *J* = 6.6 Hz, 2H), 2.90 (t, *J* = 6.9 Hz, 2H), 1.32 (s, 6H). ^{13}C NMR (101 MHz, $CDCl_3$) δ 170.80, 170.30, 147.24, 146.73, 146.62, 137.21, 135.84, 130.63, 130.19, 129.81, 128.69, 127.49, 127.17, 125.61, 123.88, 122.47, 121.04, 120.51, 120.33, 120.29, 116.62, 110.36, 33.11, 33.08, 27.95, 21.01, 20.93, 14.32. Calcd. for $C_{37}H_{32}N_6O_5$ $[M]^+$ 640.2434 found 640,2410

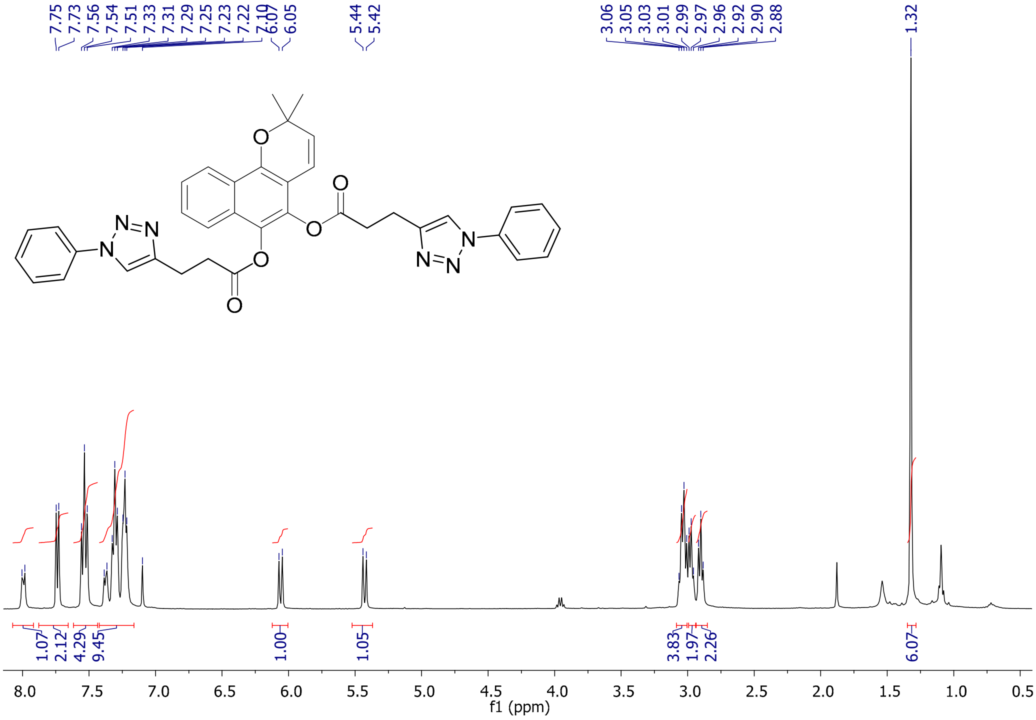

Figure S1. 400 MHz ^{1}H NMR spectra in $CDCl_3$ of **2a**.

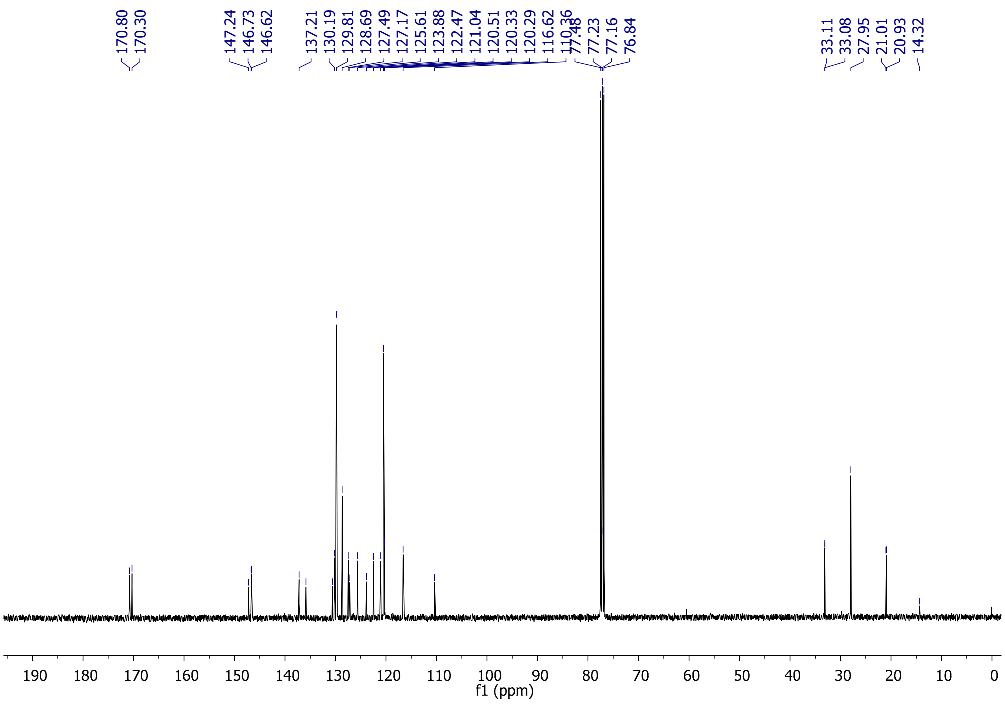

Figure S2. 100 MHz ^{13}C NMR spectra in $CDCl_3$ of **2a**.

Compound 2b. Lapachol (24.2 mg, 0.1 mmol) and 4-pentynoic acid (20 μL, 0.2 mmol), 4-azidoanisole solution (440 μL, 0.22 mmol, c = 0.5 M) were reacted in DCM:THF 1:1 vv (2 mL) according to the stepwise esterification-click general procedure. Flash column chromatography purification (n-hexane/EtOAc 6:4) afforded **2b** (40 mg, 57%) as a light yellow solid. ^{1}H NMR (400 MHz, $CDCl_3$) δ 8.16 (d, *J* = 8.6 Hz, 1H), 7.81 (d, *J* = 8.0 Hz, 2H), 7.57 (dd, *J* = 20.8, 11.6 Hz, 5H), 7.44 – 7.31 (m, 2H), 6.96 (d, *J* = 8.1 Hz, 3H), 6.23 (d, *J* = 9.9 Hz, 1H), 5.61 (d, *J* = 9.9 Hz, 1H), 3.84 (s, 5H), 3.22 – 3.15 (m, 4H), 3.12 (t, *J* = 6.5 Hz, 2H), 3.05 (t, *J* = 6.9 Hz, 2H), 1.50 (s, 6H). ^{13}C NMR (101 MHz, $CDCl_3$) δ 170.85, 170.34, 159.82, 146.54, 146.43, 130.74, 130.20, 127.50, 127.20, 125.63, 122.49, 122.17, 121.08, 120.51, 116.68, 114.85, 110.40, 55.74, 33.14, 29.84, 27.99, 21.03, 20.95. Calcd. for $C_{39}H_{36}N_6O_7$ $[M]^+$ 700.2645 found 700.2275.

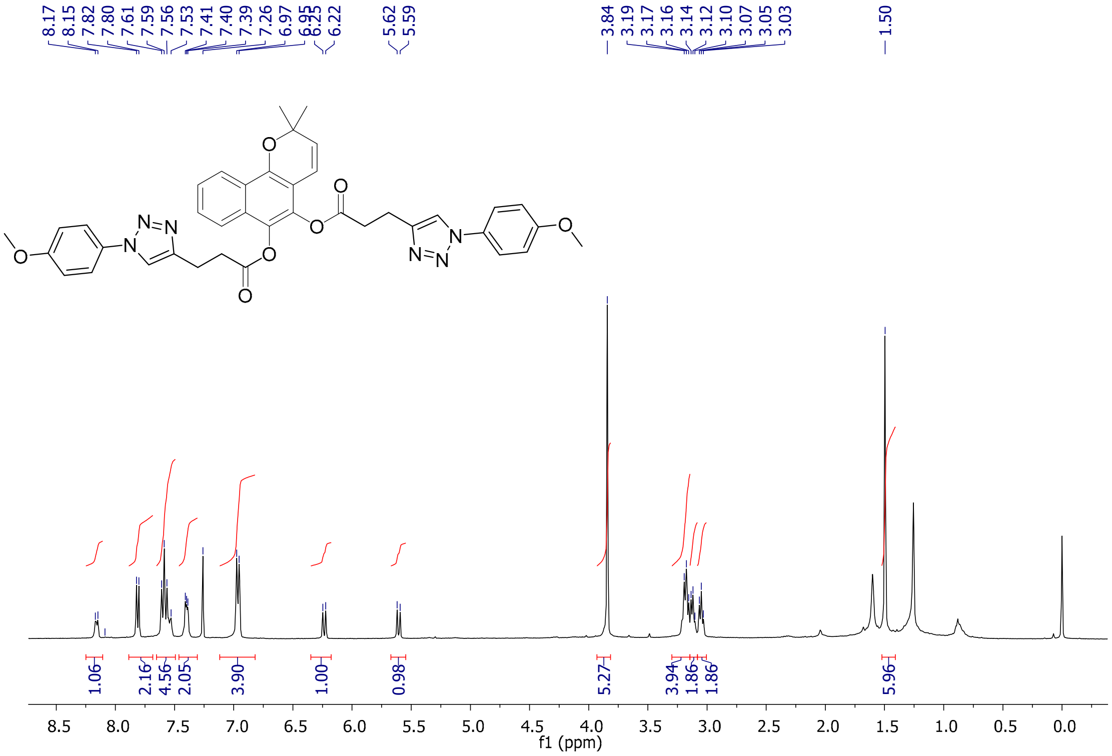

Figure S3. 400 MHz ^{1}H NMR spectra in $CDCl_3$ of **2b**.

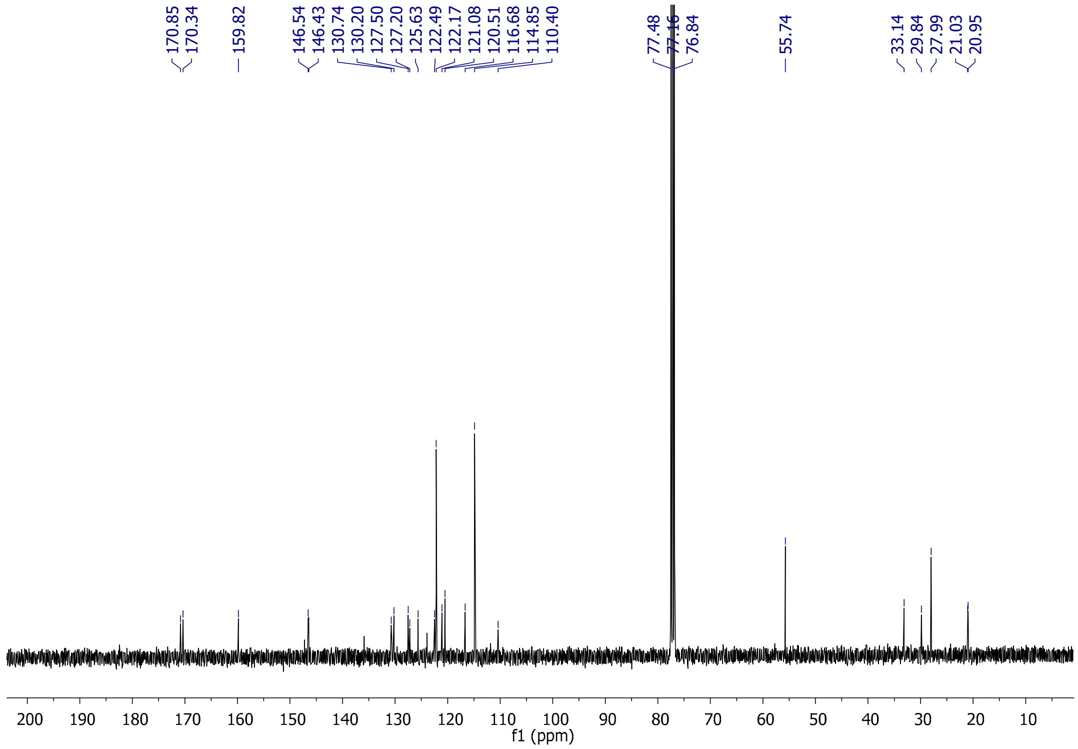

Figure S4. 100 MHz ^{13}C NMR spectra in $CDCl_3$ of **2b**.

Compound 2c. Lapachol (24.2 mg, 0.1 mmol) and 4-pentynoic acid (20 μL, 0.2 mmol), 1-Azido-4-chlorobenzene solution (440 μL, 0.22 mmol, c = 0.5 M) were reacted in DCM:THF 1:1 vv (2 mL) according to the stepwise esterification-click general procedure. Flash column chromatography purification (n-hexane/EtOAc 6:4) afforded **2c** (53 mg, 75%) as a light yellow solid. ^{1}H NMR (400 MHz, $CDCl_3$) δ 8.16 (d, *J* = 8.0 Hz, 1H), 7.87 (d, *J* = 9.8 Hz, 2H), 7.65 (t, *J* = 9.3 Hz, 4H), 7.58 – 7.37 (m, 7H), 6.23 (d, *J* = 9.9 Hz, 1H), 5.61 (d, *J* = 9.9 Hz, 1H), 3.17 (m, 4H), 3.14 – 3.10 (m, 2H), 3.05 (t, *J* = 6.8 Hz, 2H), 1.50 (s, 6H). ^{13}C NMR (101 MHz, $CDCl_3$) δ 170.69, 170.27, 146.88, 135.70, 134.52, 130.26, 130.03, 127.54, 127.16, 125.69, 122.54, 121.68, 121.02, 120.27, 116.62, 77.48, 77.29, 77.16, 76.84, 33.01, 28.00, 20.97, 20.89. Calcd. for $C_{37}H_{30}Cl_2N_6O_5$ $[M]^+$ 708.1655 found 708.1607

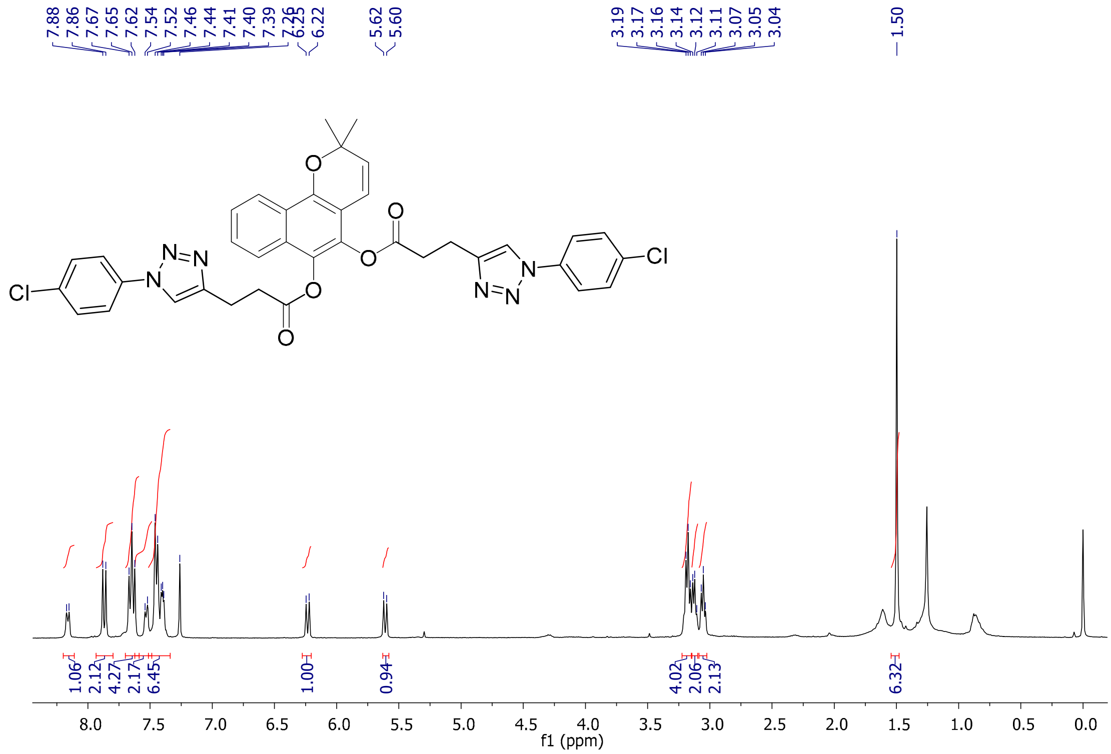

Figure S5. 400 MHz ^{1}H NMR spectra in $CDCl_3$ of **2c**.

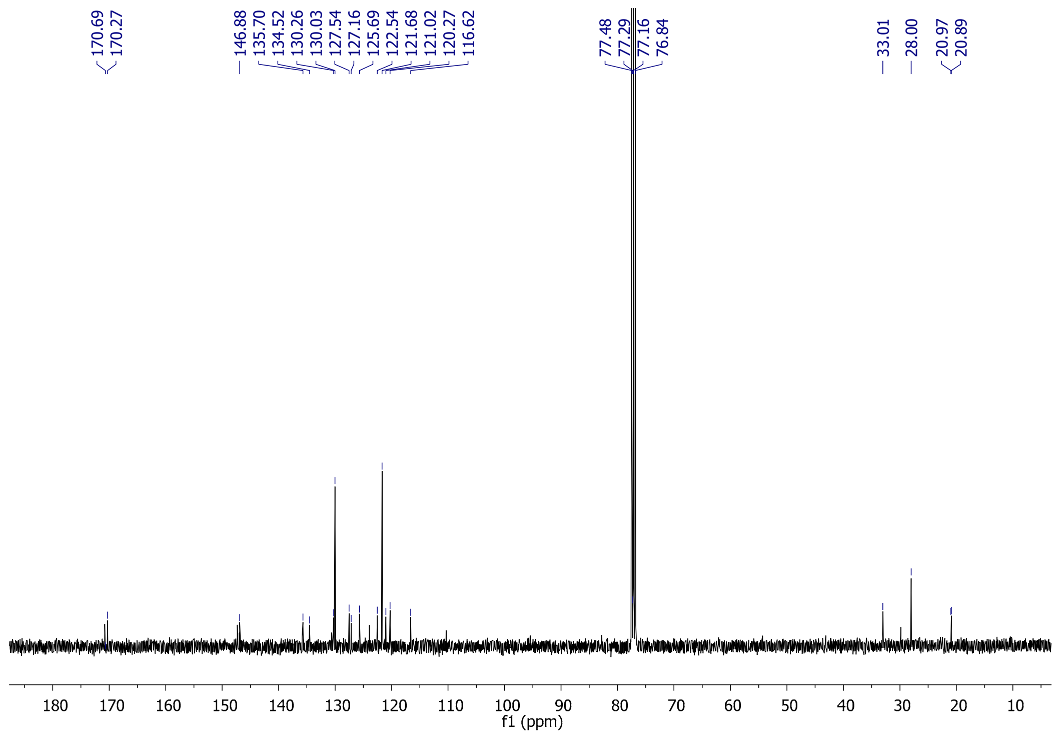

Figure S6. 100 MHz ^{13}C NMR spectra in $CDCl_3$ of **2c**.

Compound 2d. Lapachol (24.2 mg, 0.1 mmol) and 4-pentynoic acid (20 μL, 0.2 mmol), azidotoluene solution (440 μL, 0.22 mmol, *c* = 0.5 M) were reacted in DCM:THF 1:1 *vv* (2 mL) according to the stepwise esterification-click general procedure. Flash column chromatography purification (*n*-hexane/EtOAc 6:4) afforded **2d** (47 mg, 70%) as a light yellow solid. ^{1}H NMR (400 MHz, $CDCl_3$) δ 8.23 – 8.09 (m, 1H), 7.85 (d, *J* = 7.2 Hz, 2H), 7.56 (t, *J* = 8.4 Hz, 5H), 7.46 – 7.36 (m, 2H), 7.25 (d, *J* = 7.3 Hz, 4H), 6.22 (d, *J* = 9.9 Hz, 1H), 5.59 (d, *J* = 9.9 Hz, 1H), 3.18 (m, 4H), 3.12 (t, *J* = 6.7 Hz, 2H), 3.05 (t, *J* = 6.9 Hz, 2H), 2.39 (s, 6H), 1.49 (s, 6H). ^{13}C NMR (101 MHz, $CDCl_3$) δ 170.82, 170.32, 147.23, 146.57, 146.47, 138.75, 135.85, 134.95, 130.64, 130.29, 130.18, 127.48, 127.18, 125.60, 123.88, 122.46, 121.06, 120.42, 120.31, 120.28, 116.64, 110.38, 33.14, 33.11, 27.96, 21.19, 21.01, 20.94. Calcd. for $C_{39}H_{36}N_6O_5$ $[M]^+$ 668.2747 found 668.2721

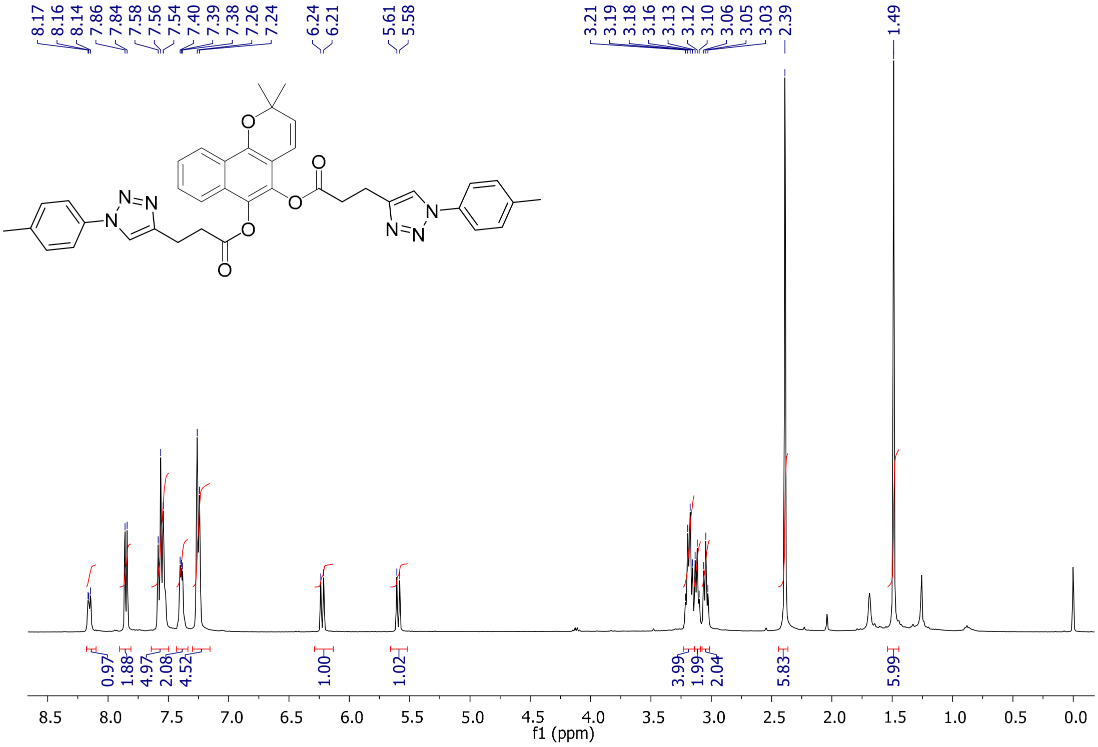

Figure S7. 400 MHz ^{1}H NMR spectra in $CDCl_3$ of **2d**.

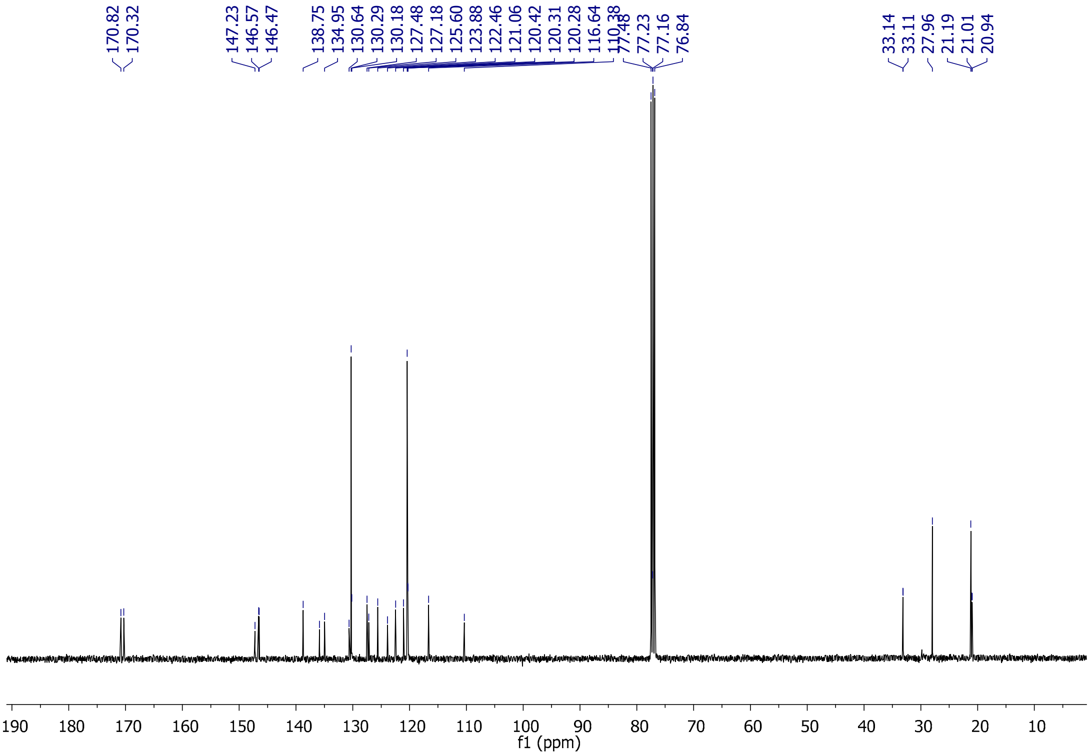

Figure S8. 100 MHz ^{13}C NMR spectra in $CDCl_3$ of **2d**.

Compound **2e**. Lapachol (24.2 mg, 0.1 mmol) and 4-pentynoic acid (20 μL, 0.2 mmol), 2,3,4-tri-O-acetyl-β-D-xylopyranosyl azide (66 mg, 0.22 mmol) were reacted in DCM:THF 1:1 *vv* (2 mL) according to the stepwise esterification-click general procedure. Flash column chromatography purification (*n*-hexane/EtOAc 6:4) afforded **2e** (57 mg, 57%) as a white solid. ^{1}H NMR (400 MHz, $CDCl_3$) δ 8.15 (d, *J* = 7.5 Hz, 1H), 7.72 (d, *J* = 4.0 Hz, 2H), 7.57 (d, *J* = 8.1 Hz, 1H), 7.47 – 7.35 (m, 2H), 6.28 (d, *J* = 9.9 Hz, 1H), 5.82 – 5.71 (m, 2H), 5.65 (d, *J* = 9.9 Hz, 1H), 5.47 – 5.35 (m, 4H), 5.21 – 5.09 (m, 2H), 4.27 (dt, *J* = 12.0, 6.1 Hz, 2H), 3.56 (dd, *J* = 18.8, 10.8 Hz, 2H), 3.13 (d, *J* = 6.1 Hz, 6H), 2.98 (d, *J* = 7.3 Hz, 2H), 2.06 (s, 6H), 2.04 (s, 6H), 1.84 (s, 3H), 1.82 (s, 3H), 1.50 (s, 6H). ^{13}C NMR (101 MHz, $CDCl_3$) δ 170.36, 169.92, 169.86, 169.72, 169.00, 147.04, 146.66, 146.56, 135.68, 130.49, 130.05, 127.33, 127.07, 125.45, 123.72, 122.29, 120.99, 120.17, 116.61, 110.29, 86.20, 86.17, 72.20, 70.32, 68.44, 65.47, 53.44, 32.80, 27.81, 20.84, 20.76, 20.63, 20.58, 20.14, 20.09. Calcd. for $C_{47}H_{52}N_6O_{19}$ $[M]^+$ 1004.3287 found 1004.3264

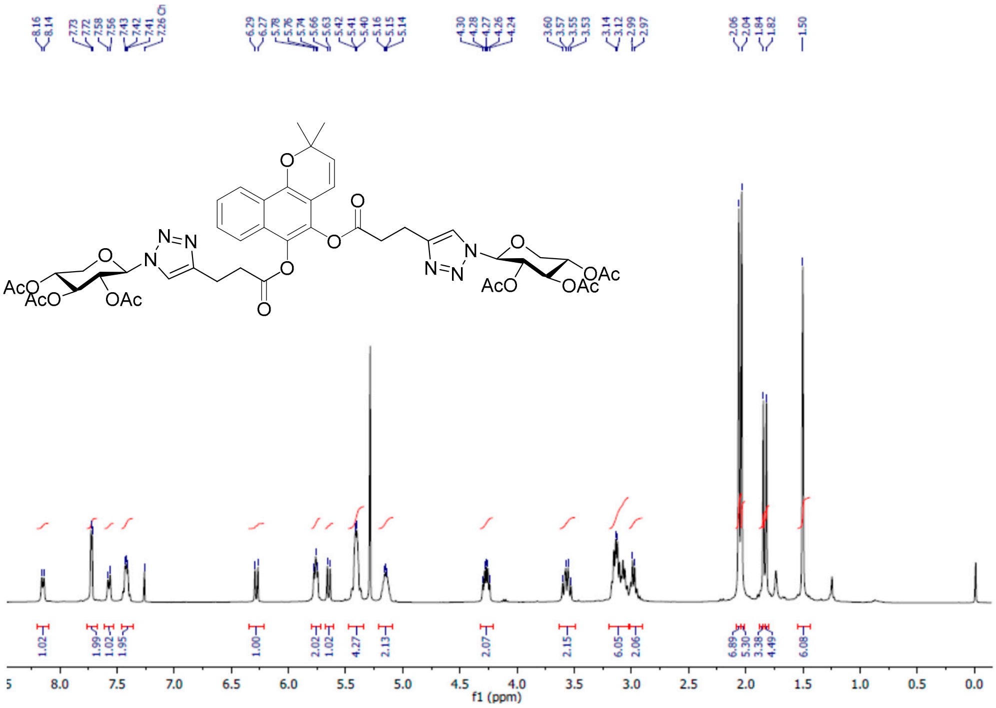

Figure S9. 400 MHz ^{1}H NMR spectra in $CDCl_3$ of **2e**.

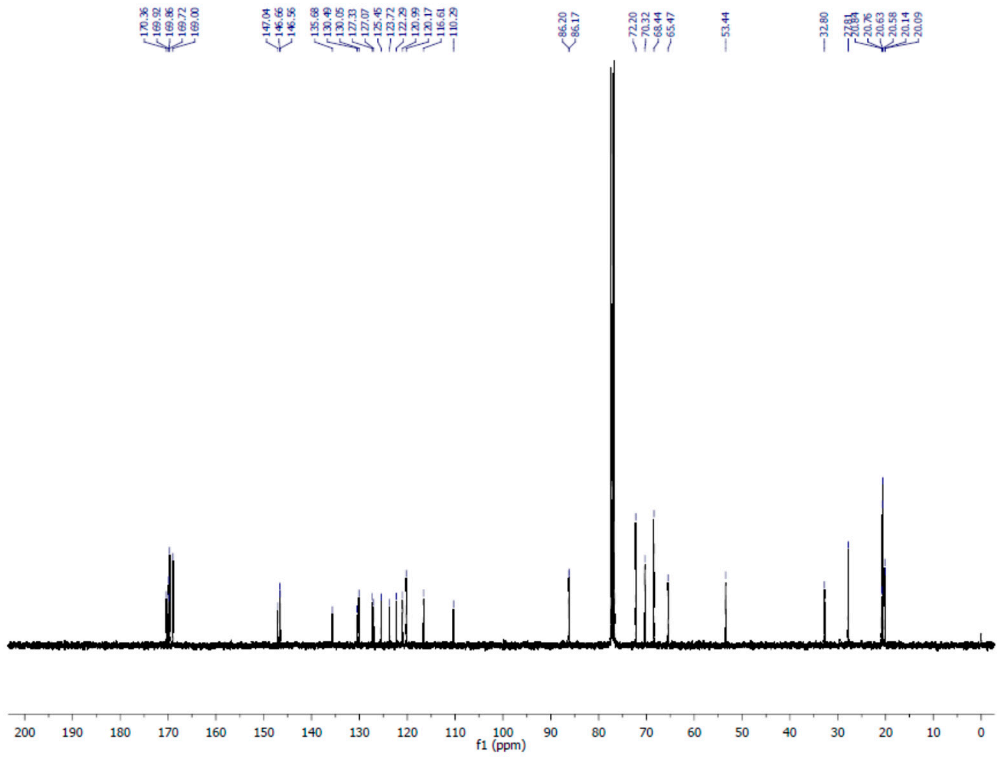

Figure S10. 100 MHz ^{13}C NMR spectra in $CDCl_3$ of **2e**.

Compound 2f. Lapachol (24.2 mg, 0.1 mmol) and 4-pentynoic acid (20 µL, 0.2 mmol), p-Toluenesulfonyl azide (440 µL, 0.22 mmol, c = 0.5 M) were reacted in DCM:THF 1:1 *vv* (2 mL) according to the stepwise esterification-click general procedure. Flash column chromatography purification (*n*-hexane/EtOAc 6:4) afforded **2f** (54 mg, 68%) as a light yellow solid. ^{1}H NMR (400 MHz, $CDCl_3$) δ 8.20 – 8.09 (m, 1H), 8.03 (s, 2H), 8.00 – 7.89 (m, 4H), 7.52 – 7.42 (m, 1H), 7.41 – 7.34 (m, 2H), 7.30 (dd, J = 7.9, 2.8 Hz, 4H), 6.16 (d, J = 9.9 Hz, 1H), 5.62 (d, J = 9.9 Hz, 1H), 5.27 (s, 1H), 3.11 – 3.02 (m, 4H), 3.00 (t, J = 6.9 Hz, 2H), 2.93 (t, J = 7.0 Hz, 2H), 2.39 (s, 5H), 1.48 (s, 6H), 1.22 (s, 2H). ^{13}C NMR (101 MHz, $CDCl_3$) δ 170.30, 169.82, 147.35, 145.94, 145.83, 133.30, 130.57, 130.33, 128.79, 127.55, 125.67, 123.89, 122.50, 121.83, 120.95, 116.52, 110.33, 53.52, 32.37, 27.97, 21.95, 20.71, 20.64. Calcd. for $C_{39}H_{36}N_6O_9S_2$ $[M]^+$ 796.1985 found 796.1962

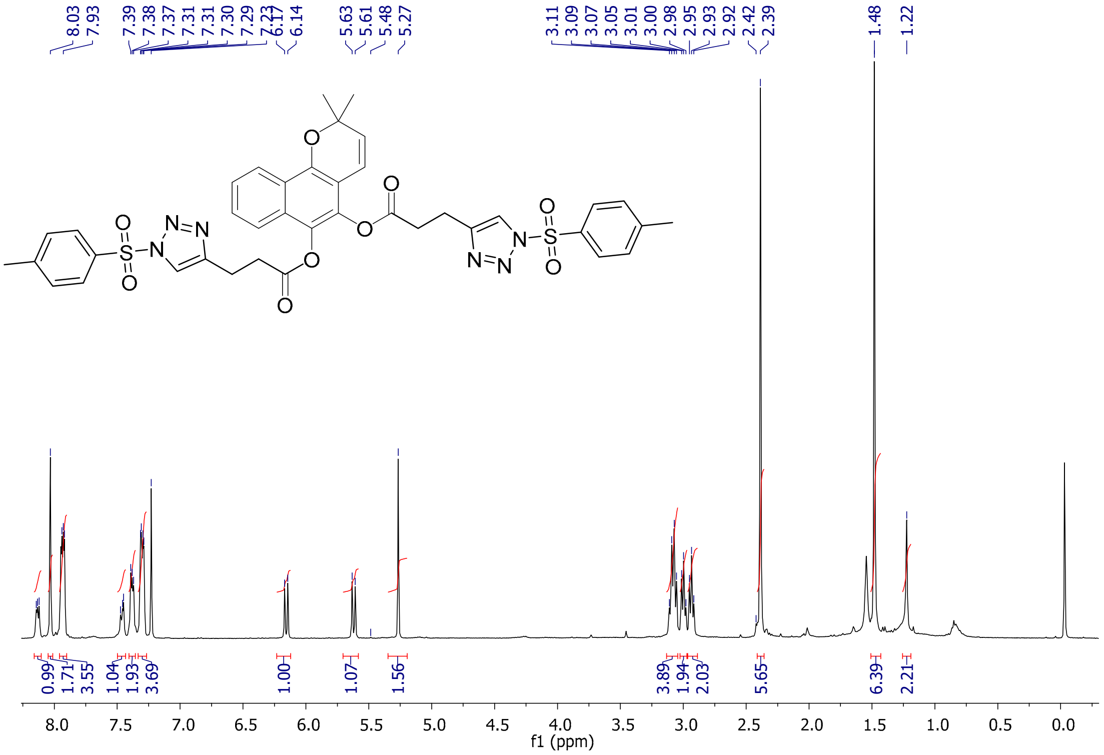

Figure S11. 400 MHz ^{1}H NMR spectra in $CDCl_3$ of **2f**.

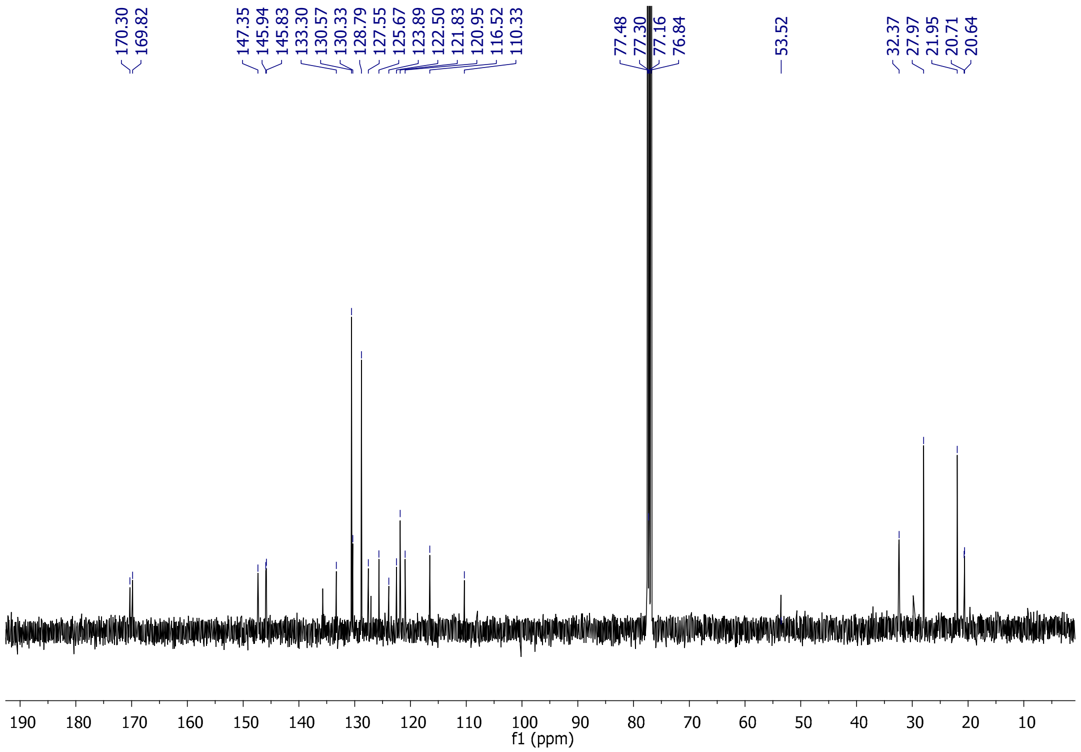

Figure S12. 100 MHz ^{13}C NMR spectra in $CDCl_3$ of **2f**.

Compound 2g. Lapachol (24.2 mg, 0.1 mmol) and 4-pentynoic acid (20 μL, 0.2 mmol), 1-Azido-3-chlorobenzene solution (440 μL, 0.22 mmol, *c* = 0.5 M) were reacted in DCM:THF 1:1 *vv* (2 mL) according to the stepwise esterification-click general procedure. Flash column chromatography purification (*n*-hexane/EtOAc 6:4) afforded **2g** (48 mg, 68%) as a light yellow solid. ^1H NMR (400 MHz, $CDCl_3$) δ 8.07 – 7.94 (m, 1H), 7.75 (d, *J* = 8.5 Hz, 2H), 7.60 (d, *J* = 8.5 Hz, 2H), 7.50 – 7.36 (m, 3H), 7.23 (t, *J* = 9.4 Hz, 6H), 6.07 (d, *J* = 9.9 Hz, 1H), 5.45 (d, *J* = 9.9 Hz, 1H), 3.05 (m, 4H), 2.99 (t, *J* = 6.8 Hz, 2H), 2.92 (t, *J* = 6.8 Hz, 2H), 1.34 (s, 6H). ^{13}C NMR (101 MHz, $CDCl_3$) δ 170.74, 170.23, 147.26, 146.97, 146.87, 138.00, 135.79, 135.62, 130.91, 130.90, 130.56, 130.22, 128.74, 127.51, 127.12, 125.65, 123.87, 122.49, 120.98, 120.70, 120.68, 120.26, 120.23, 118.43, 116.56, 110.32, 60.50, 32.97, 32.95, 29.80, 27.95, 21.15, 20.94, 20.86, 14.31. Calcd. for $C_{37}H_{30}Cl_2N_6O_5$ $[M]^+$ 708.1655 found 708.1679

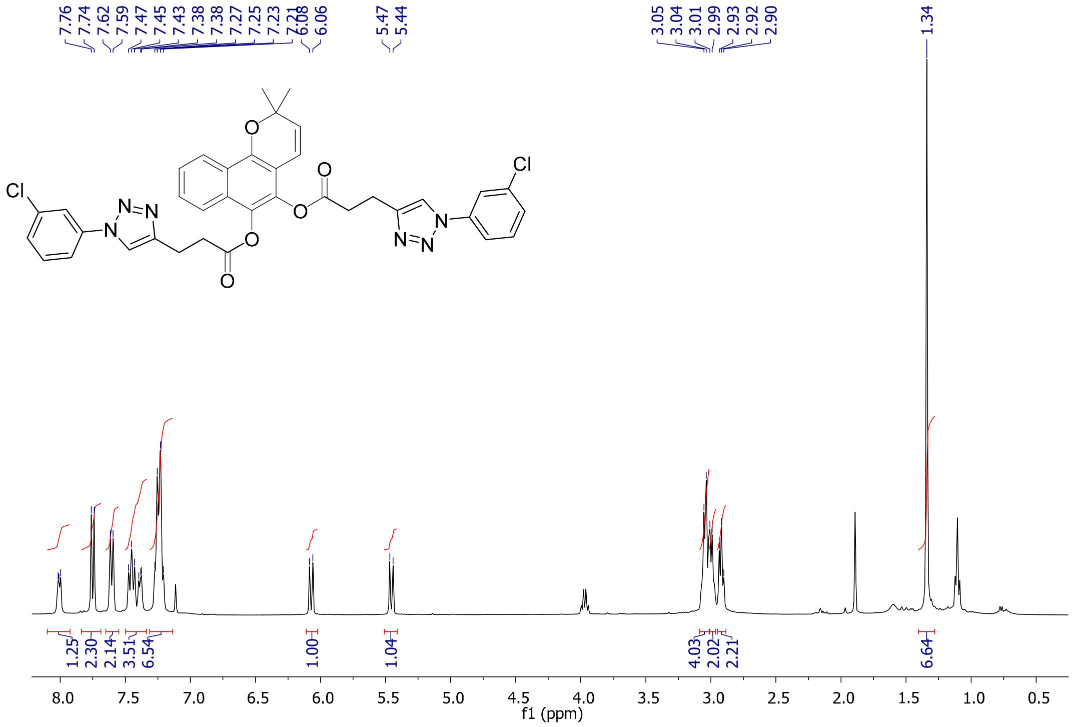

Figure S13. 400 MHz ^1H NMR spectra in $CDCl_3$ of **2g**.

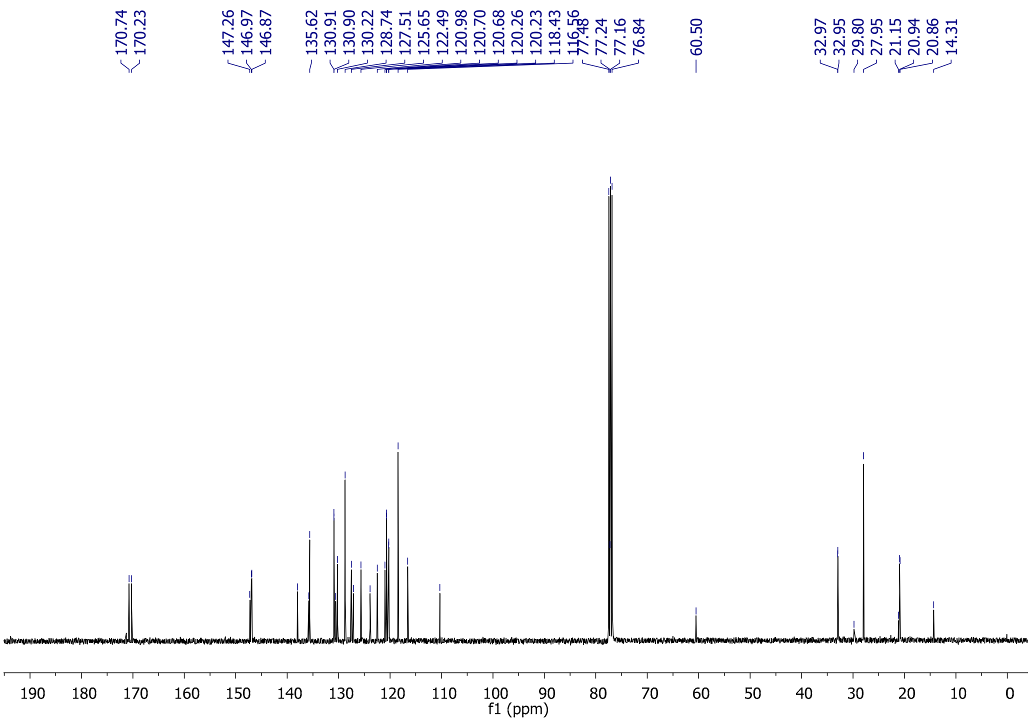

Figure S14. 100 MHz ^{13}C NMR spectra in $CDCl_3$ of **2g**.

Compound 2h. Lapachol (24.2 mg, 0.1 mmol) and 4-pentynoic acid (20 μL, 0.2 mmol), 1-(azidomethyl)-4-nitrobenzene solution (440 μL, 0.22 mmol, *c* = 0.5 M) were reacted in DCM:THF 1:1 *vv* (2 mL) according to the stepwise esterification-click general procedure. Flash column chromatography purification (*n*-hexane/EtOAc 6:4) afforded **2h** (57 mg, 75%) as a light yellow solid. ^1H NMR (400 MHz, $CDCl_3$) δ 8.22 – 7.99 (m, 5H), 7.69 – 7.16 (m, 9H), 6.13 (d, *J* = 9.9 Hz, 1H), 5.54 (d, *J* = 9.9 Hz, 1H), 5.47 (s, 2H), 5.46 (s, 2H), 3.01 (m, 4H), 2.95 (t, *J* = 6.5 Hz, 2H), 2.89 (t, *J* = 6.8 Hz, 2H), 1.40 (s, 6H). ^{13}C NMR (100 MHz, $CDCl_3$) δ 170.68, 170.18, 148.08, 147.27, 146.80, 146.71, 141.98, 141.94, 135.74, 130.47, 130.36, 128.60, 128.58, 127.55, 127.07, 125.70, 124.34, 124.32, 123.84, 122.56, 122.38, 120.91, 116.50, 110.32, 60.51, 53.06, 32.95, 32.92, 27.93, 21.16, 20.97, 20.87, 14.31. Calcd. for $C_{39}H_{34}N_8O_9$ $[M]^+$ 758.2449 found 758.2496

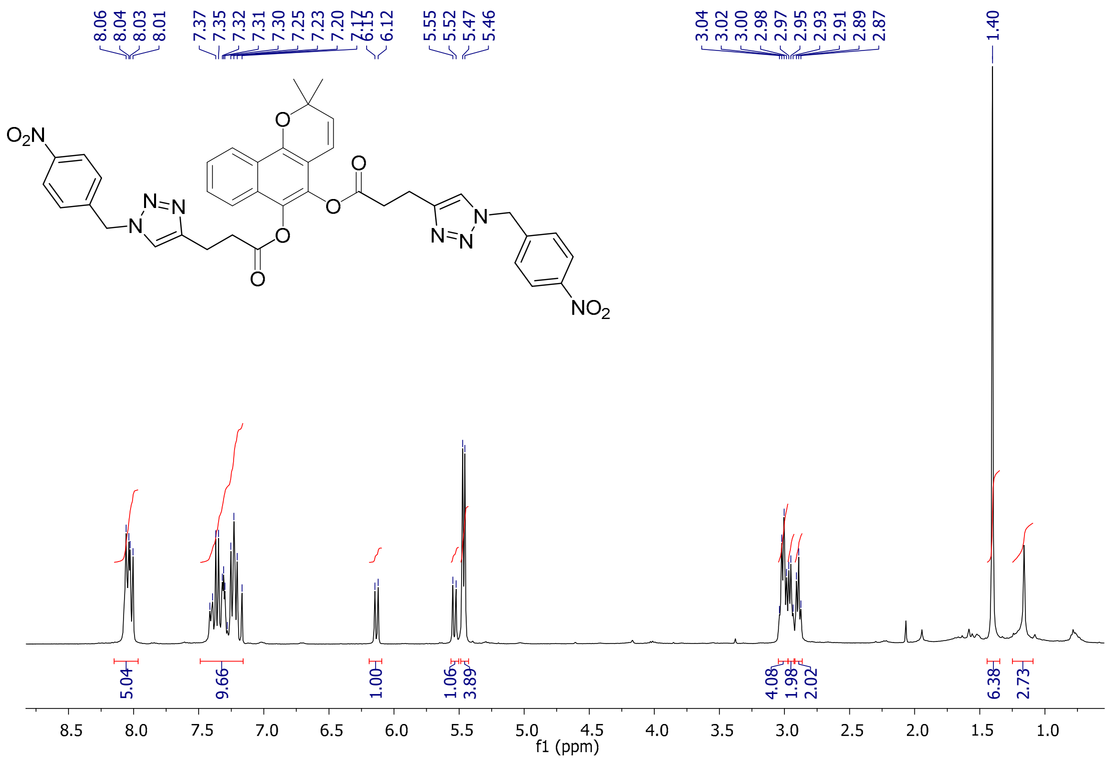

Figure S15. 400 MHz ^{1}H NMR spectra in $CDCl_3$ of **2h**.

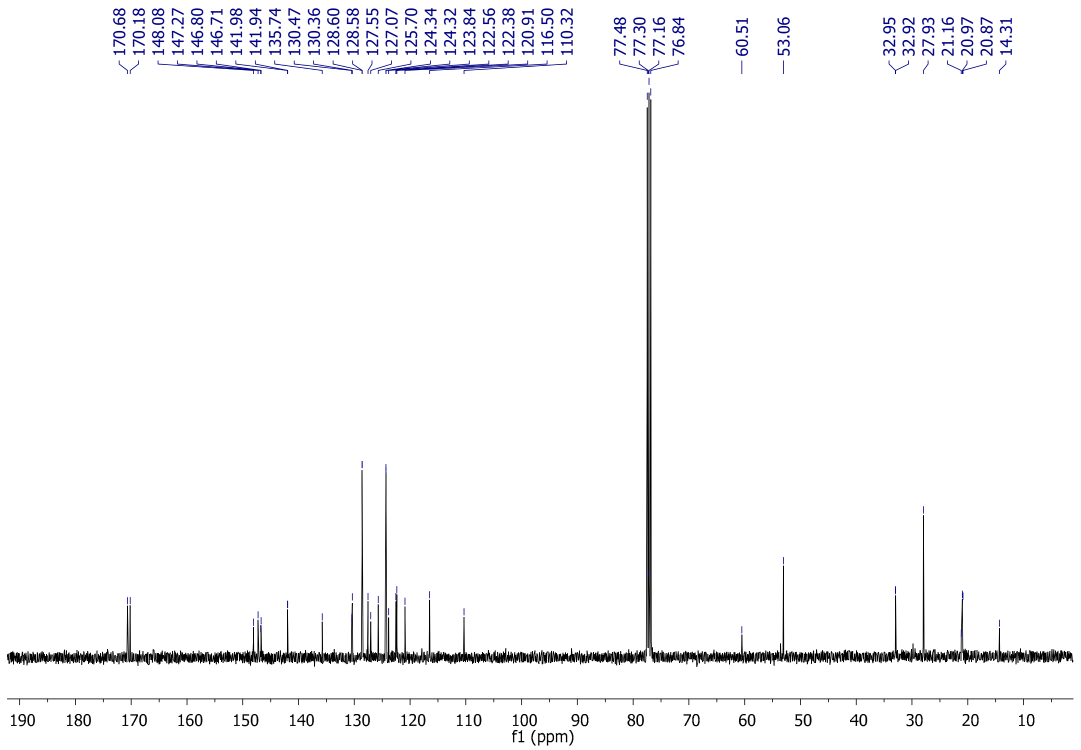

Figure S16. 100 MHz ^{13}C NMR spectra in $CDCl_3$ of **2h**.

Compound 2i. Lapachol (24.2 mg, 0.1 mmol) and 4-pentynoic acid (20 μL, 0.2 mmol), 1-(azidomethyl)-4-bromobenzene solution (440 μL, 0.22 mmol, *c* = 0.5 M) were reacted in DCM:THF 1:1 *vv* (2 mL) according to the stepwise esterification-click general procedure. Flash column chromatography purification (*n*-hexane/EtOAc 6:4) afforded **2i** (62 mg, 75%) as a light yellow solid. ^{1}H NMR (400 MHz, $CDCl_3$) δ 7.95 (d, *J* = 7.8 Hz, 1H), 7.23 (m, 8H), 6.88 (t, *J* = 8.2 Hz, 4H), 6.01 (d, *J* = 9.9 Hz, 1H), 5.40 (d, *J* = 9.9 Hz, 1H), 5.19 (s, 2H), 5.18 (s, 2H), 2.88 (dd, *J* = 15.5, 7.3 Hz, 4H), 2.80 (t, *J* = 6.9 Hz, 2H), 2.74 (t, *J* = 7.0 Hz, 2H), 1.30 (s, 6H). ^{13}C NMR (100 MHz, $CDCl_3$) δ 170.70, 170.20, 147.23, 146.58, 146.49, 135.78, 133.99, 133.96, 132.36, 132.34, 130.53, 130.22, 129.72, 127.49, 127.14, 125.64, 123.86, 122.90, 122.50, 121.99, 121.95, 121.00, 116.62, 110.36, 53.43, 33.03, 29.82, 27.99, 21.02, 20.92. Calcd. for $C_{39}H_{34}Br_2N_6O_5$ $[M]^+$ 824.0957 found 824.0978

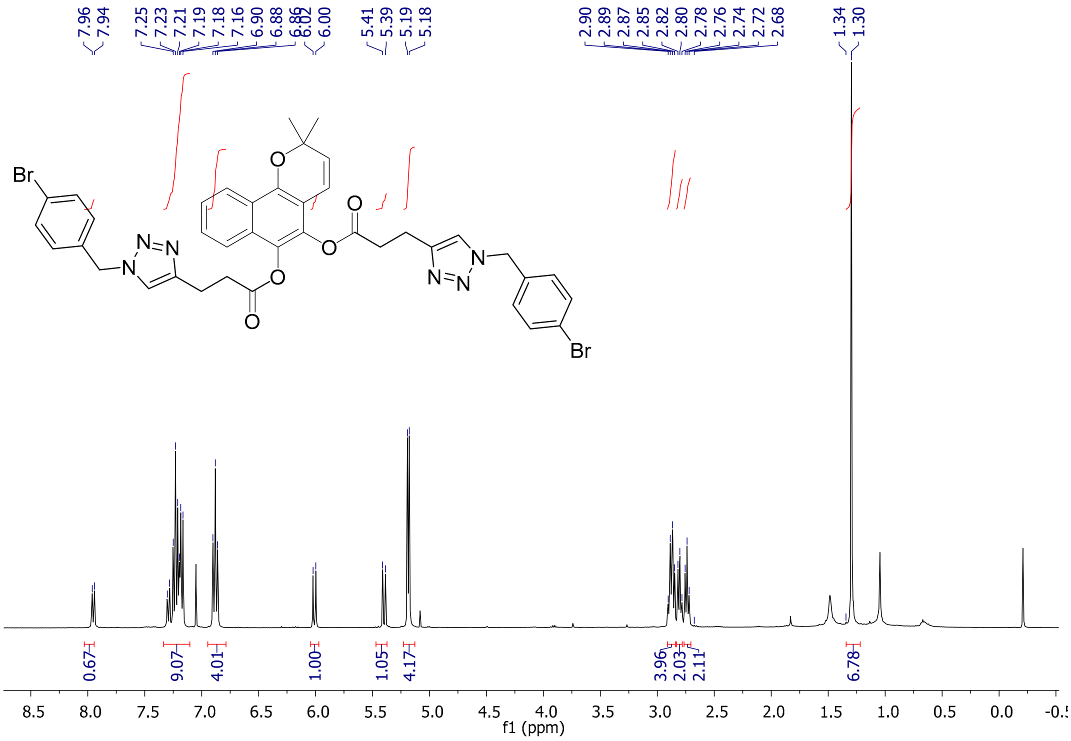

Figure S17. 400 MHz ^{1}H NMR spectra in $CDCl_3$ of **2i**.

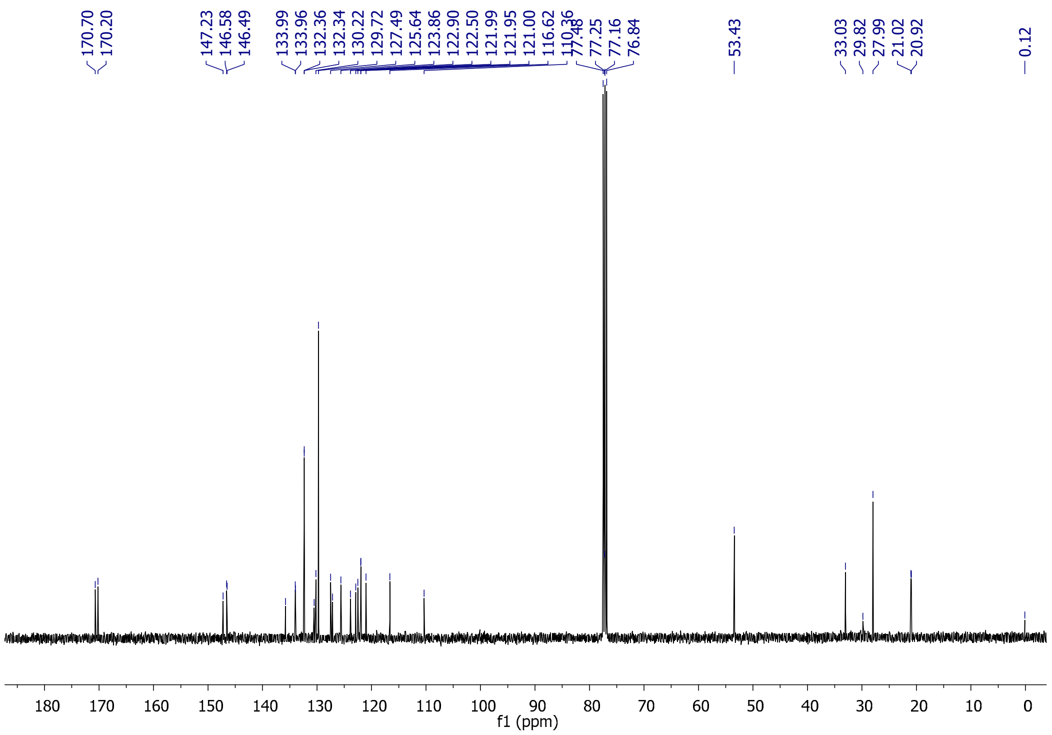

Figure S18. 100 MHz ^{13}C NMR spectra in $CDCl_3$ of **2i**.

Compound 3a. Lapachol (24.2 mg, 0.1 mmol) and 5-hexynoic acid (22 μL, 0.2 mmol), azidobenzene solution (440 μL, 0.22 mmol, *c* = 0.5 M) were reacted in DCM:THF 1:1 *vv* (2 mL) according to the stepwise esterification-click general procedure. Flash column chromatography purification (*n*-hexane/EtOAc 6:4) afforded **3a** (55 mg, 82%) as a light yellow solid. ^{1}H NMR (400 MHz, $CDCl_3$) δ 8.18 (d, *J* = 7.5 Hz, 1H), 7.84 (s, 2H), 7.71 (d, *J* = 7.6 Hz, 4H), 7.64 (d, *J* = 8.0 Hz, 1H), 7.45 (dt, *J* = 16.9, 7.7 Hz, 8H), 6.33 (d, *J* = 9.9 Hz, 1H), 5.66 (d, *J* = 9.9 Hz, 1H), 2.96 (dd, *J* = 15.5, 7.7 Hz, 4H), 2.82 (t, *J* = 7.2 Hz, 2H), 2.76 (t, *J* = 7.2 Hz, 2H), 2.23 (dt, *J* = 21.2, 7.1 Hz, 4H), 1.52 (s, 6H). ^{13}C NMR (101 MHz, $CDCl_3$) δ 171.21, 170.69, 147.71, 147.66, 147.21, 137.27, 135.91, 130.69, 130.23, 129.82, 128.63, 127.48, 127.28, 125.58, 123.89, 122.51, 121.06, 120.49, 119.54, 116.73, 110.48, 33.20, 33.17, 27.96, 24.99, 24.94, 24.80, 24.68. Calcd. for $C_{39}H_{36}N_6O_5$ $[M]^+$ 668.2747 found 668.2740.

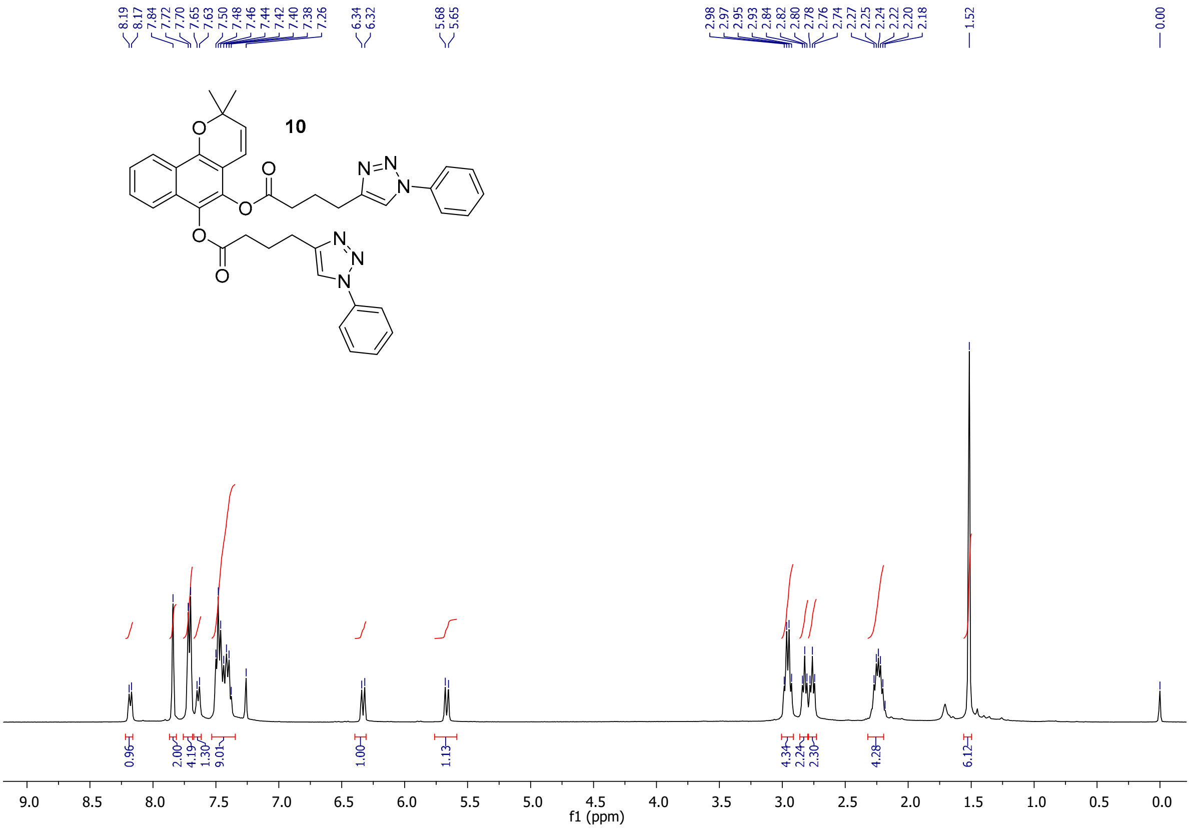

Figure S19. 400 MHz ^{1}H NMR spectra in $CDCl_3$ of **3a**.

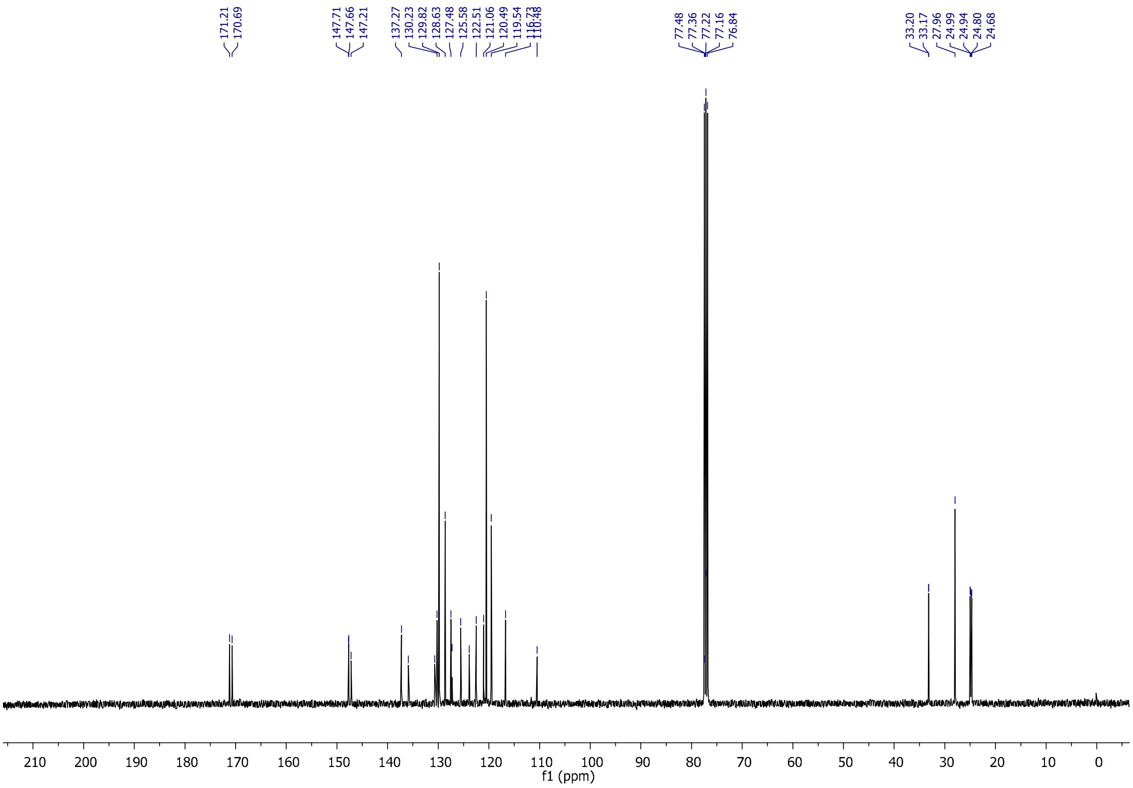

Figure S20. 100 MHz ^{13}C NMR spectra in $CDCl_3$ of **3a**.

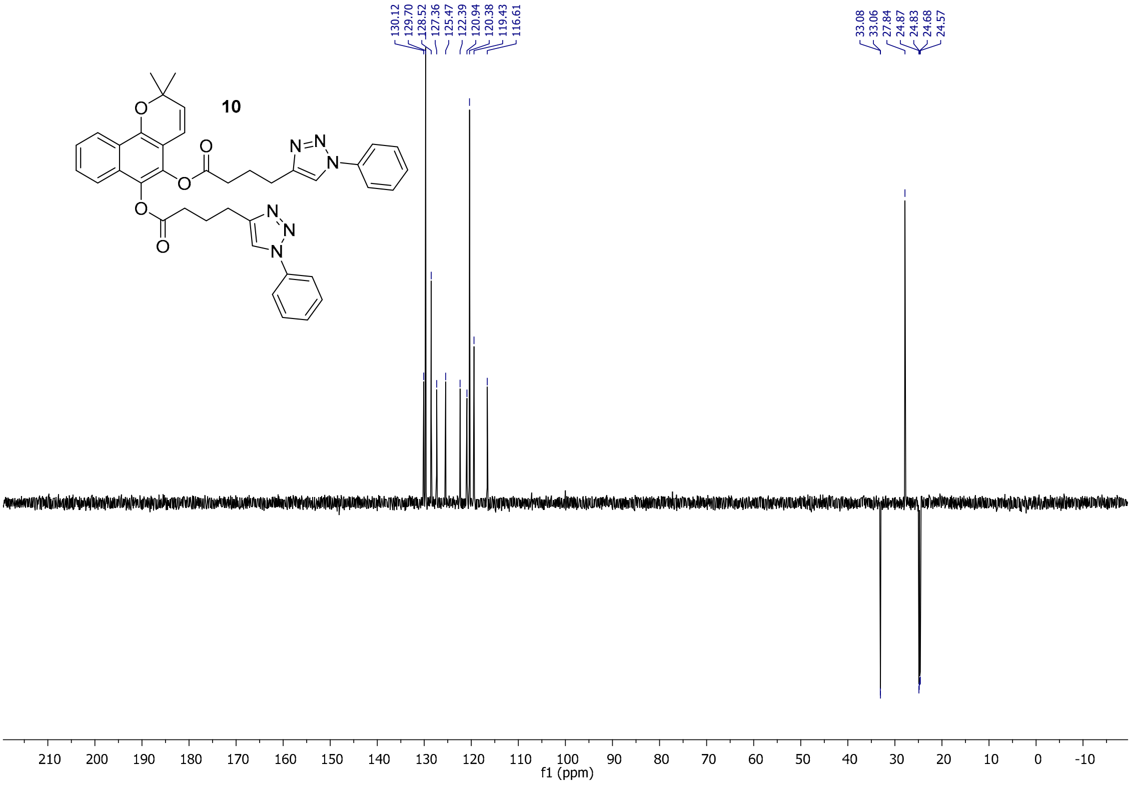

Figure S21. DEPT 135 spectra in $CDCl_3$ of **3a**.

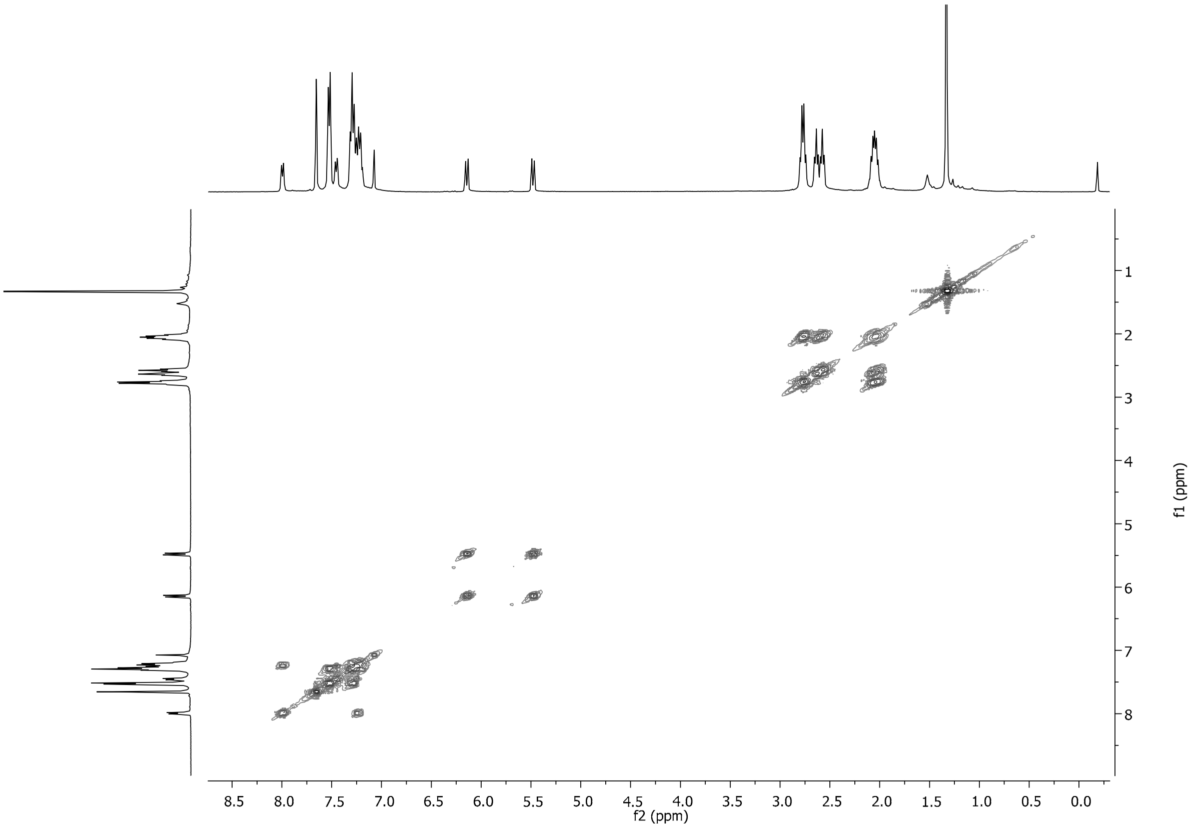

Figure S22. COSY spectra in $CDCl_3$ of **3a**.

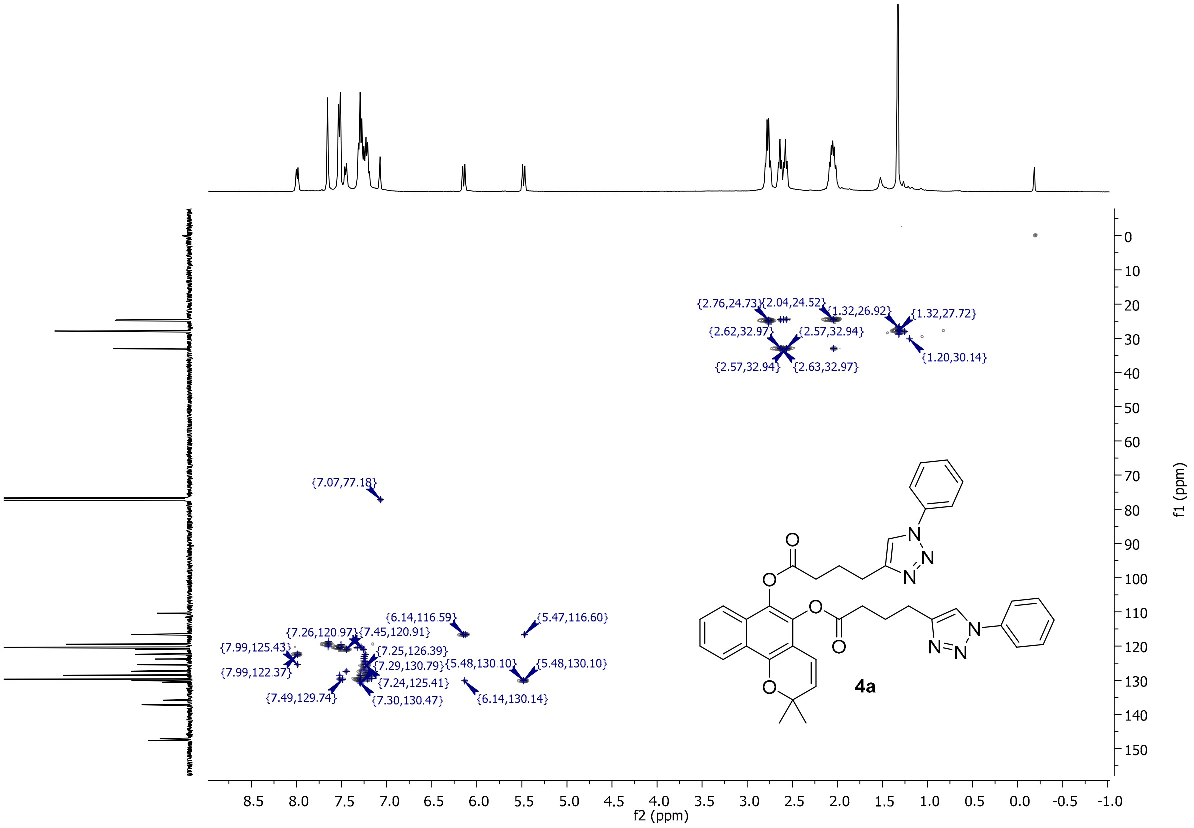

Figure S23. HSQC spectra in $CDCl_3$ of **3a**.

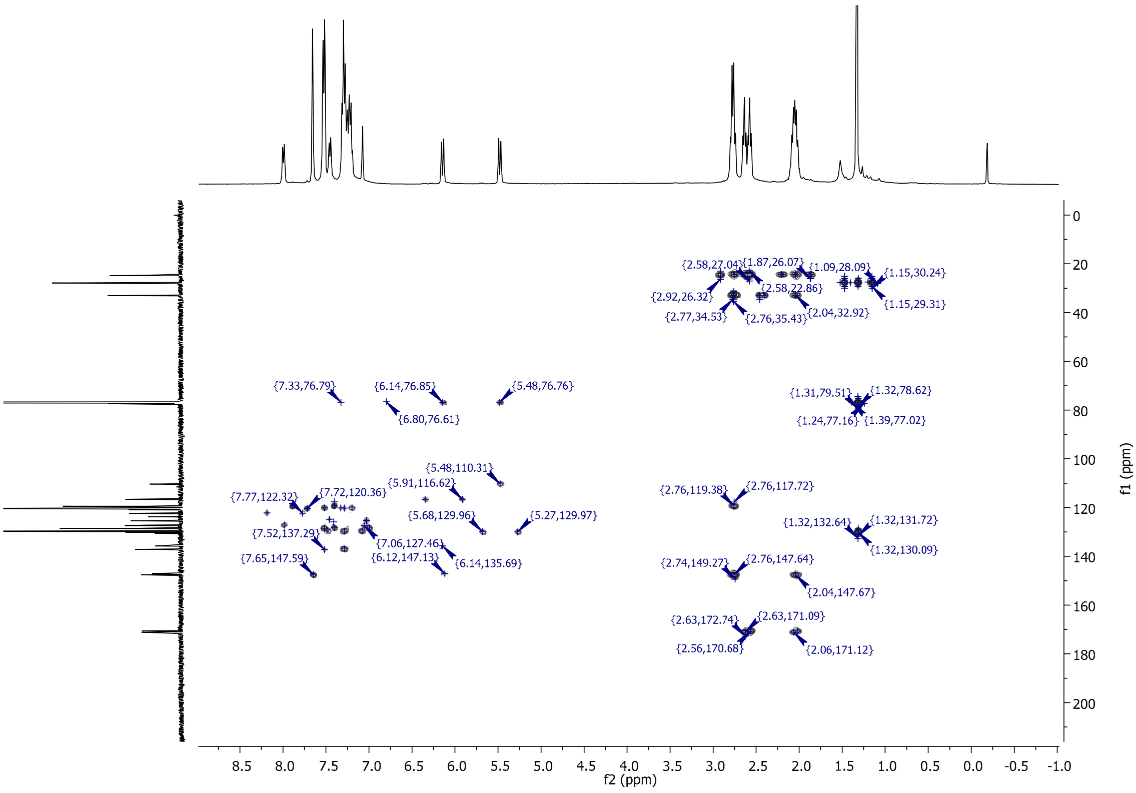

Figure S24. HMBC spectra in $CDCl_3$ of **3a**.

Compound 3b. Lapachol (24.2 mg, 0.1 mmol) and 5-hexynoic acid (22 μL, 0.2 mmol), 4-azidoanisole solution (440 μL, 0.22 mmol, *c* = 0.5 M) were reacted in DCM:THF 1:1 *vv* (2 mL) according to the stepwise esterification-click general procedure. Flash column chromatography purification (*n*-hexane/EtOAc 6:4) afforded **3b** (37 mg, 51%) as a light yellow solid. ^{1}H NMR (400 MHz, $CDCl_3$) δ 8.18 (d, *J* = 7.8 Hz, 1H), 7.75 (s, 2H), 7.63 (d, *J* = 7.7 Hz, 2H), 7.60 (d, *J* = 8.6 Hz, 4H), 7.44 (dt, *J* = 13.2, 6.7 Hz, 2H), 6.98 (d, *J* = 8.9 Hz, 4H), 6.33 (d, *J* = 9.9 Hz, 1H), 5.67 (d, *J* = 9.9 Hz, 1H), 3.84 (s, 6H), 2.94 (m, 4H), 2.81 (t, *J* = 7.4 Hz, 2H), 2.75 (t, *J* = 7.4 Hz, 2H), 2.23 (m, 4H), 1.52 (s, 6H). ^{13}C NMR (100 MHz, $CDCl_3$) δ 171.14, 170.63, 147.37, 147.32, 147.09, 135.78, 130.64, 130.56, 130.12, 127.36, 127.15, 125.47, 123.77, 122.39, 122.03, 120.94, 119.63, 116.61, 114.73, 110.37, 55.60, 33.10, 33.07, 27.83, 24.87, 24.82, 24.71, 24.60. Calcd. for $C_{41}H_{40}N_6O_7$ $[M]^+$ 728.2958 found 728.2927.

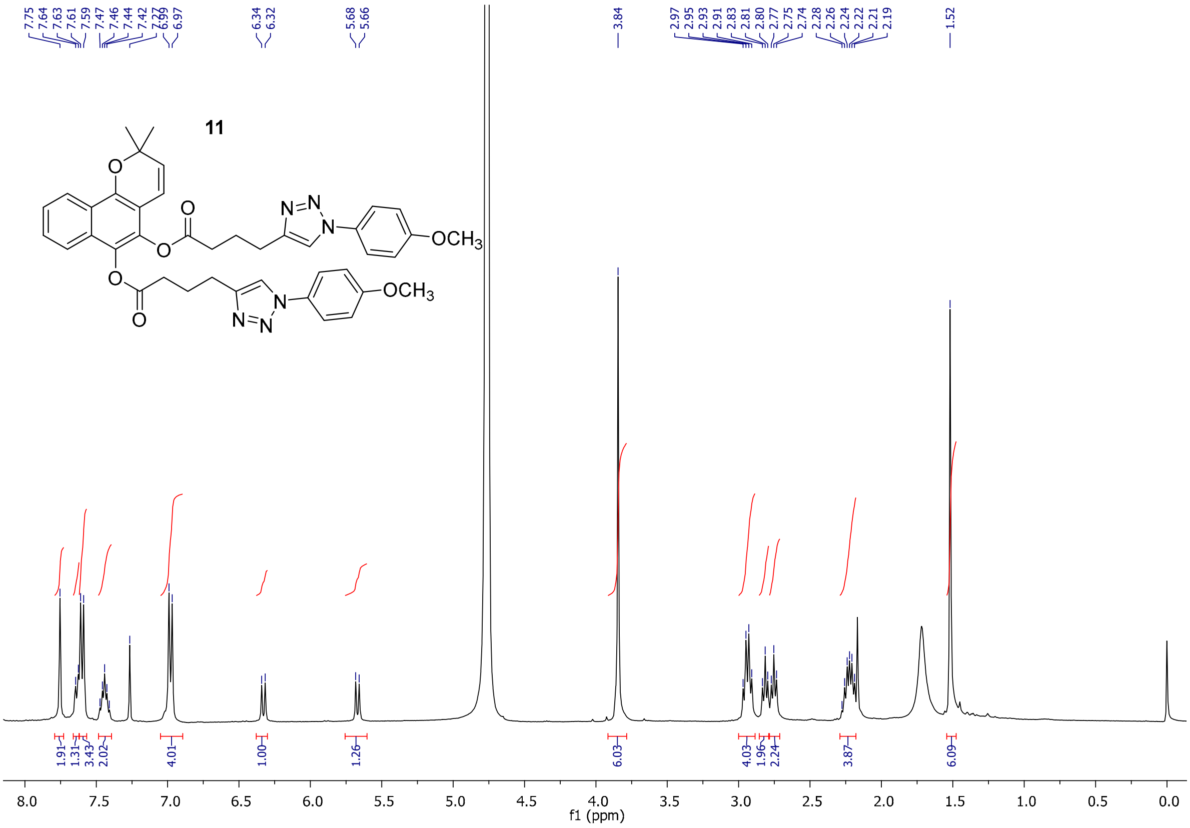

Figure S25. 400 MHz ^{1}H NMR spectra in $CDCl_3$ of **3b**.

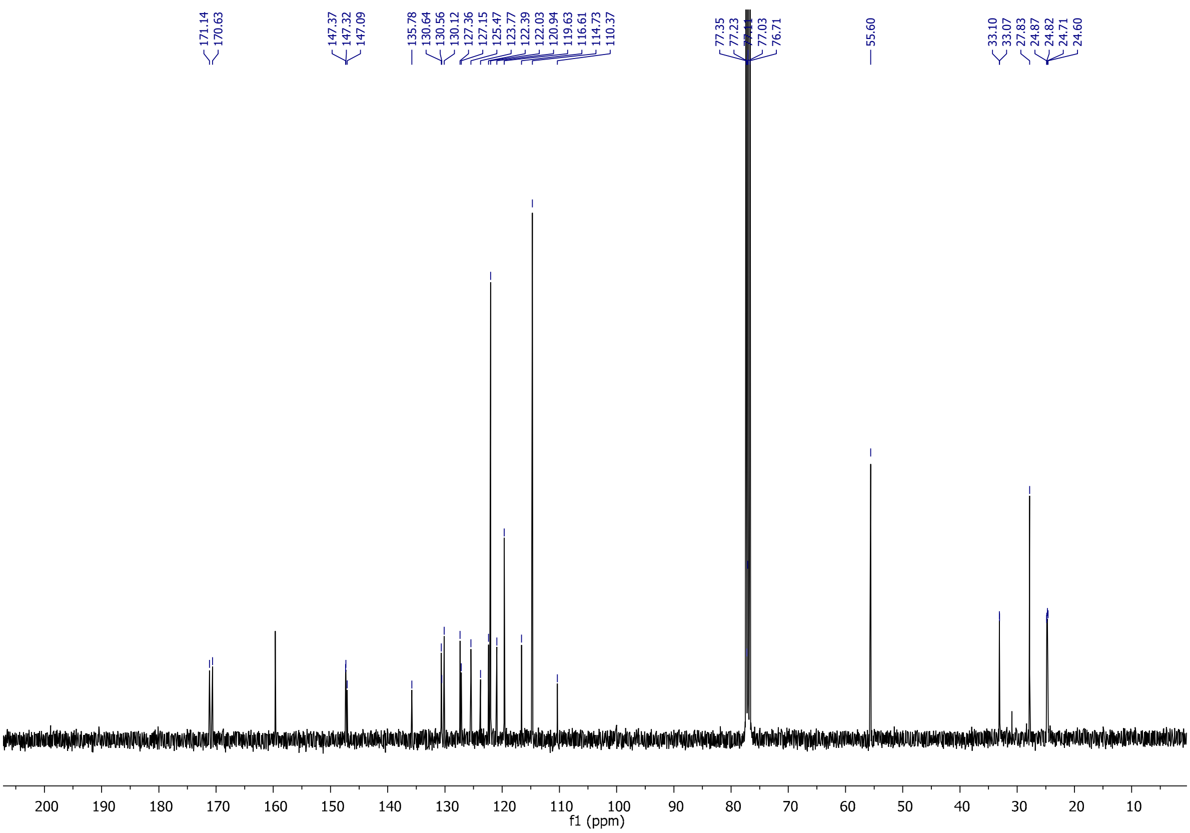

Figure S26. 100 MHz ^{13}C NMR spectra in $CDCl_3$ of **3b**.

Compound 3c. Lapachol (24.2 mg, 0.1 mmol) and 5-hexynoic acid (22 μL, 0.2 mmol), 1-Azido-4-chlorobenzene solution (440 μL, 0.22 mmol, *c* = 0.5 M) were reacted in DCM:THF 1:1 *vv* (2 mL) according to the stepwise esterification-click general procedure. Flash column chromatography purification (*n*-hexane/EtOAc 6:4) afforded **3c** (60 mg, 81%) as a light yellow solid. ^{1}H NMR (400 MHz, $CDCl_3$) δ 8.17 (d, *J* = 7.8 Hz, 1H), 7.82 (s, 2H), 7.65 (d, *J* = 8.7 Hz, 4H), 7.64 (d, *J* = 7.8 Hz, 1H), 7.45 (d, *J* = 7.6 Hz, 4H), 7.43 (m, 2H), 6.32 (d, *J* = 9.9 Hz, 1H), 5.67 (d, *J* = 9.9 Hz, 1H), 2.94 (q, *J* = 7.8 Hz, 4H), 2.81 (t, *J* = 7.3 Hz, 2H), 2.75 (t, *J* = 7.3 Hz, 2H), 2.30 – 2.15 (m, 4H), 1.51 (s, 6H). ^{13}C NMR (100 MHz, $CDCl_3$) δ 171.18, 170.66, 147.92, 147.24, 135.88, 135.72, 134.38, 130.65, 130.28, 129.99, 127.51, 127.24, 125.62, 123.90, 122.54, 121.62, 121.02, 119.48, 116.68, 110.45, 33.19, 33.17, 27.96, 24.97, 24.92, 24.73, 24.63. Calcd. for $C_{39}H_{34}Cl_2N_6O_5$ $[M]^+$ 736.1968 found 739.2007.

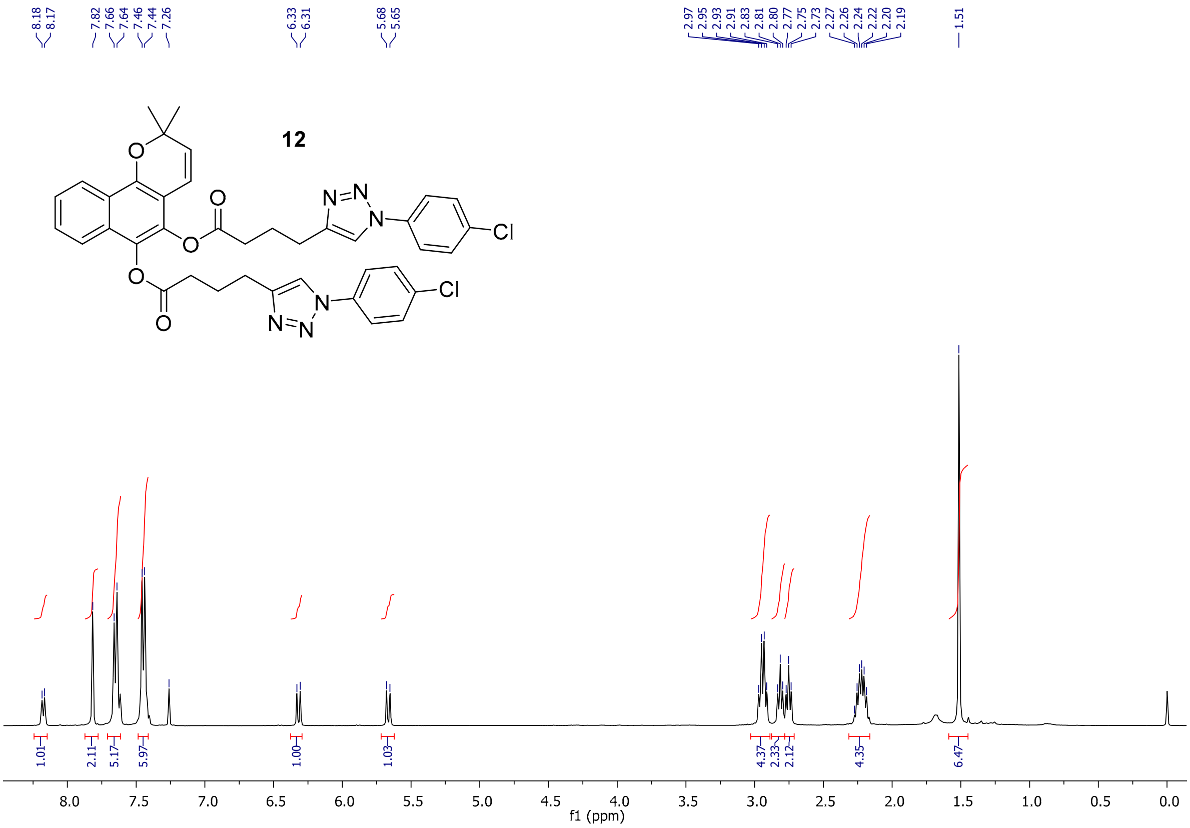

Figure S27. 400 MHz ¹H NMR spectra in $CDCl_3$ of **3c**.

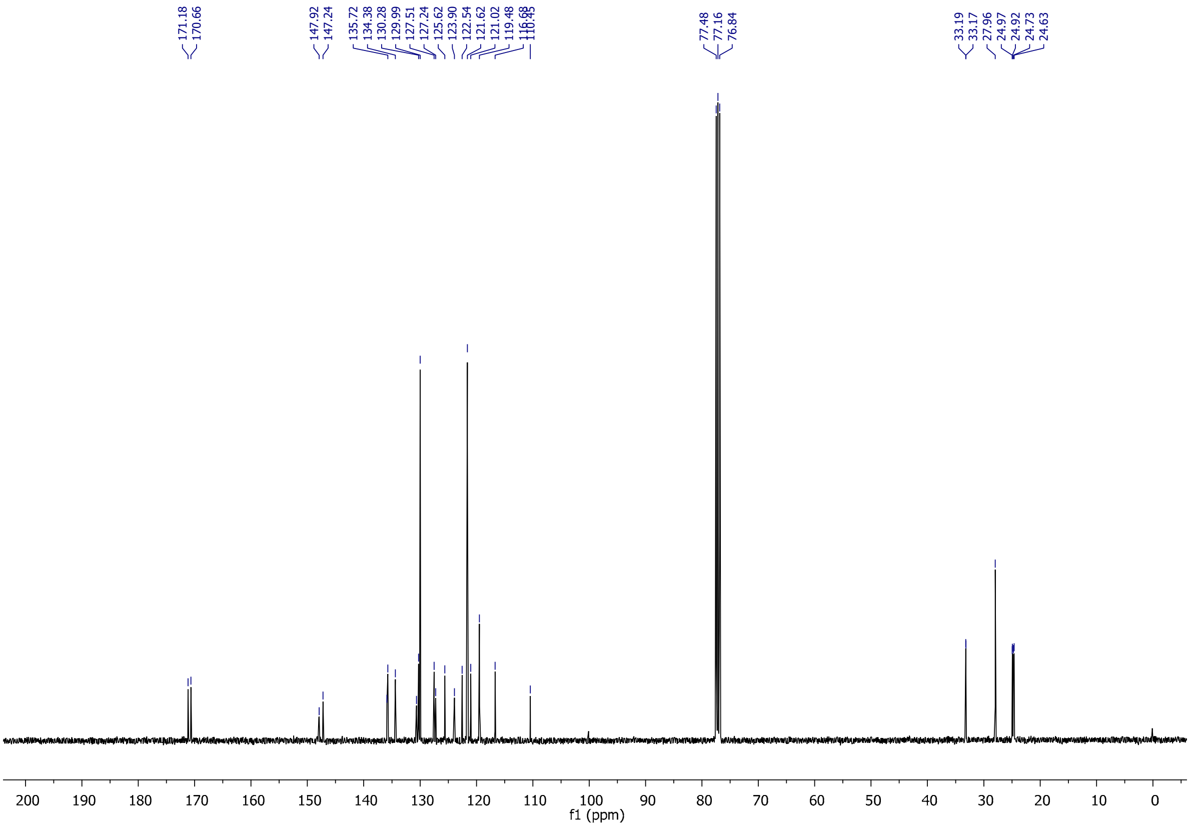

Figure S28. 100 MHz ^{13}C NMR spectra in $CDCl_3$ of **3c**.

Compound 3d. Lapachol (24.2 mg, 0.1 mmol) and 5-hexynoic acid (22 μL, 0.2 mmol), azidotoluene solution (440 μL, 0.22 mmol, *c* = 0.5 M) were reacted in DCM:THF 1:1 *vv* (2 mL) according to the stepwise esterification-click general procedure. Flash column chromatography purification (*n*-hexane/EtOAc 6:4) afforded **3d** (59 mg, 85%) as a light yellow solid. ^1H NMR (400 MHz, $CDCl_3$) δ 8.17 (d, *J* = 7.7 Hz, 1H), 7.79 (s, 2H), 7.64 (d, *J* = 7.7 Hz, 1H), 7.57 (d, *J* = 7.7 Hz, 4H), 7.49 – 7.38 (m, 2H), 7.26 (d, *J* = 7.4 Hz, 4H), 6.33 (d, *J* = 9.9 Hz, 1H), 5.66 (d, *J* = 9.9 Hz, 1H), 2.94 (q, *J* = 7.9 Hz, 4H), 2.81 (t, *J* = 7.3 Hz, 2H), 2.75 (t, *J* = 7.3 Hz, 2H), 2.39 (s, 6H), 2.23 (td, *J* = 14.0, 7.0 Hz, 4H), 1.51 (s, 6H). ^{13}C NMR (100 MHz, $CDCl_3$) δ 171.22, 170.70, 147.55, 147.50, 147.19, 138.68, 135.91, 135.00, 130.69, 130.29, 130.21, 127.46, 127.28, 125.57, 123.89, 122.50, 121.07, 120.40, 119.52, 116.74, 110.49, 33.21, 33.18, 27.96, 25.00, 24.95, 24.82, 24.71, 21.18. Calcd. for $C_{41}H_{40}N_6O_5$ $[M]^+$ 696.3060 found 696.3045.

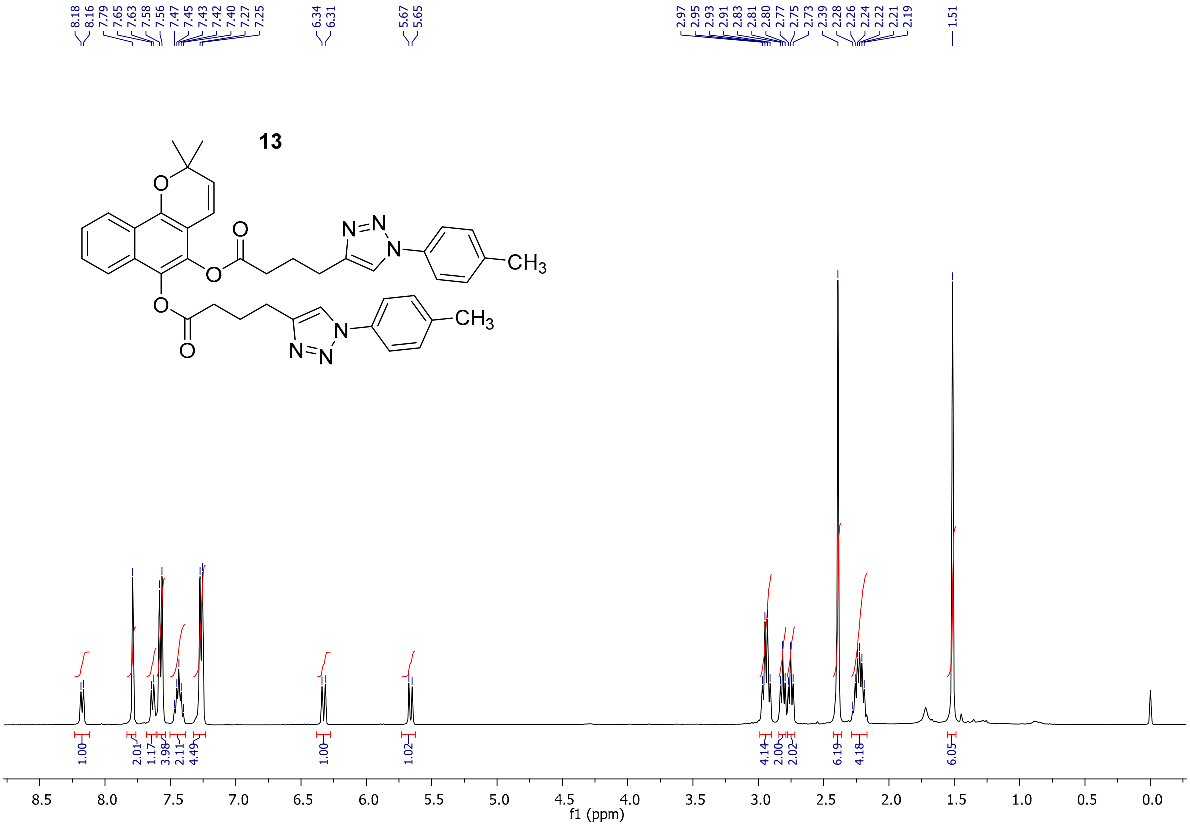

Figure S29. 400 MHz ^1H NMR spectra in $CDCl_3$ of **3d**.

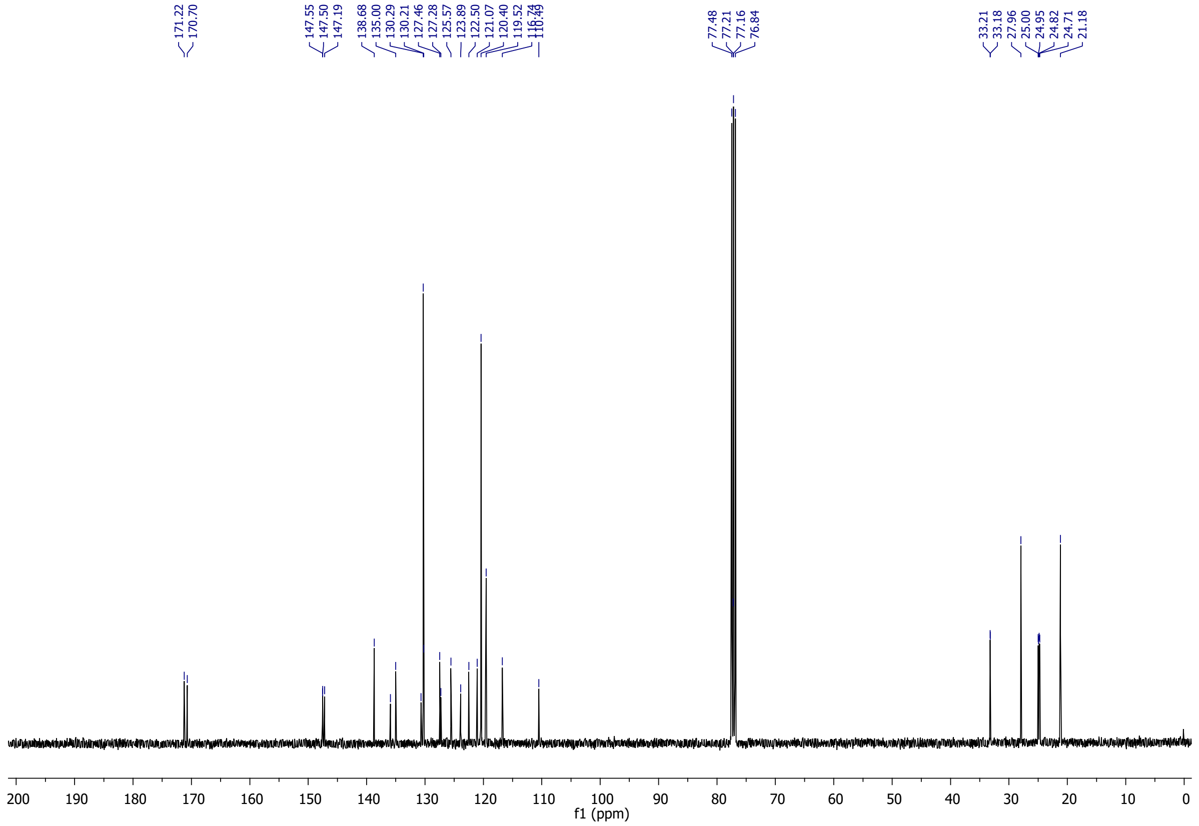

Figure S30. 100 MHz ^{13}C NMR spectra in $CDCl_3$ of **3d**.

Compound 3e. Lapachol (24.2 mg, 0.1 mmol) and 5-hexynoic acid (22 μL, 0.2 mmol), 2,3,4-tri-O-acetyl-β-D-xylopyranosyl azide (66 mg, 0.22 mmol) were reacted in DCM:THF 1:1 *vv* (2 mL) according to the stepwise esterification-click general procedure. Flash column chromatography purification (*n*-hexane/EtOAc 6:4) afforded **3e** (63 mg, 62%) as a white solid. ^{1}H NMR (400 MHz, $CDCl_3$) δ 8.20 (d, *J* = 7.9 Hz, 1H), 7.69 (s, 2H), 7.66 (d, *J* = 8.0 Hz, 1H), 7.53 – 7.41 (m, 2H), 6.35 (d, *J* = 9.8 Hz, 1H), 5.83 (d, *J* = 7.6 Hz, 2H), 5.70 (d, *J* = 9.8 Hz, 1H), 5.48 – 5.41 (m, 4H), 5.20 (dd, *J* = 14.7, 8.9 Hz, 2H), 4.31 (dd, *J* = 11.5, 5.5 Hz, 2H), 3.63 (t, *J* = 11.0 Hz, 2H), 2.91 (dd, *J* = 16.6, 7.9 Hz, 4H), 2.76 (t, *J* = 7.3 Hz, 2H), 2.70 (t, *J* = 7.3 Hz, 2H), 2.21 (tt, *J* = 14.4, 7.4 Hz, 4H), 2.10 (s, 6H), 2.08 (s, 6H), 1.90 (s, 6H), 1.54 (s, 6H). ^{13}C NMR (100 MHz, $CDCl_3$) δ 171.08, 170.56, 170.00, 169.90, 169.17, 147.72, 147.68, 147.15, 135.88, 130.68, 130.17, 127.43, 127.28, 125.53, 123.87, 122.46, 121.10, 119.60, 119.58,

116.78, 110.49, 86.41, 72.28, 70.57, 68.61, 65.66, 32.93, 32.91, 27.95, 24.91, 24.86, 24.62, 24.50, 20.75, 20.70, 20.28. Calcd. for $C_{49}H_{56}N_6O_{19}$ [M]$^+$ 1032.3600 found 1032.3597.

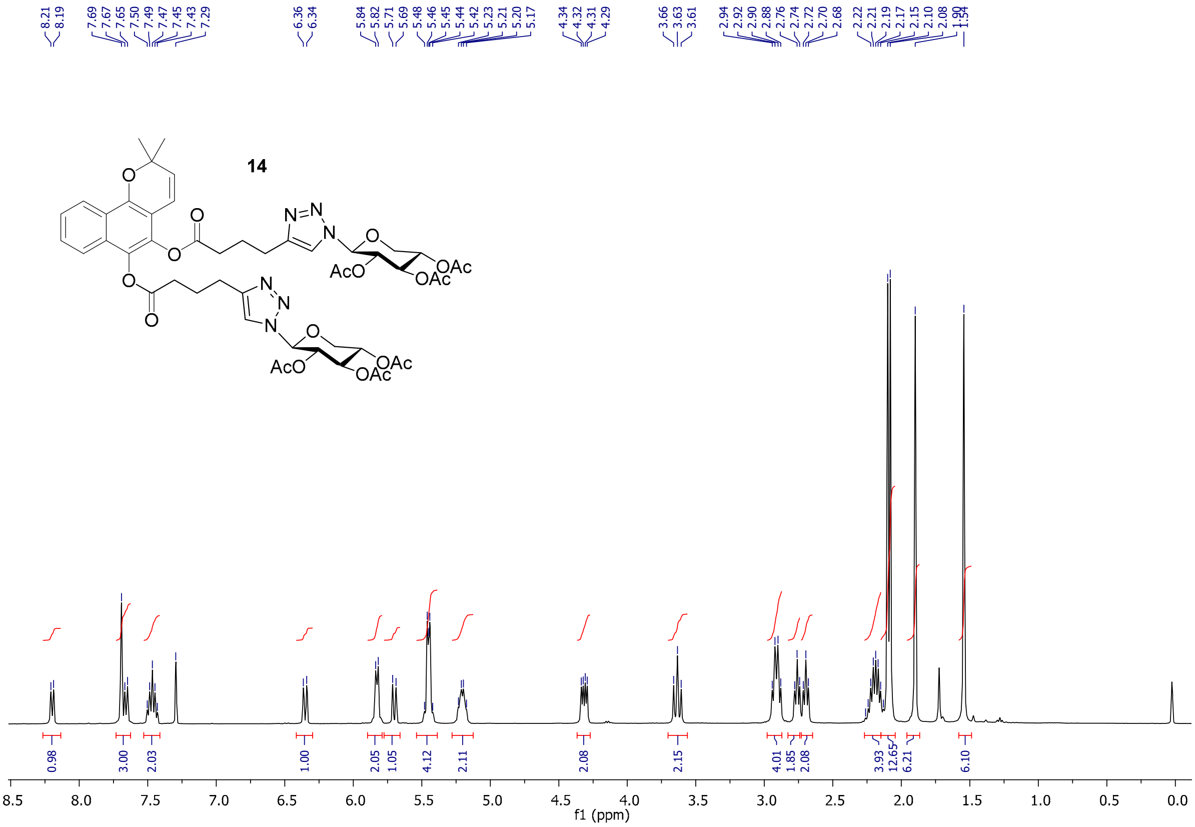

Figure S31. 400 MHz ^{1}H NMR spectra in $CDCl_3$ of **3e**.

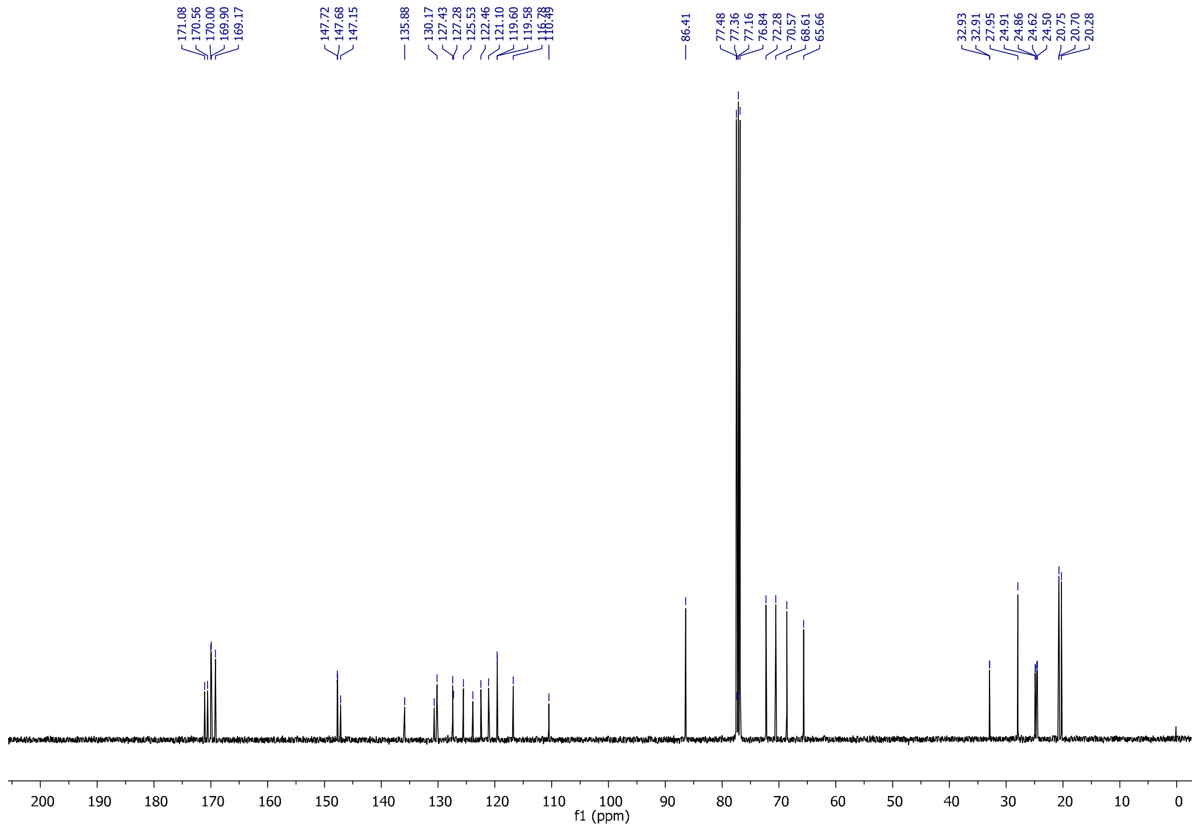

Figure S32. 100 MHz ^{13}C NMR spectra in $CDCl_3$ of **3e**.

Compound 3f. Lapachol (24.2 mg, 0.1 mmol) and 5-hexynoic acid (22 μL, 0.2 mmol), p-Toluenesulfonyl azide (440 μL, 0.22 mmol, *c* = 0.5 M) were reacted in DCM:THF 1:1 *vv* (2 mL) according to the stepwise esterification-click general procedure. Flash column chromatography purification (*n*-hexane/EtOAc 6:4) afforded **3f** (59 mg, 71%) as a light yellow solid. ^{1}H NMR (400 MHz, $CDCl_3$) δ 8.14 (d, *J* = 7.7 Hz, 1H), 8.03 – 7.88 (m, 5H), 7.55 (dd, *J* = 14.8, 7.9 Hz, 1H), 7.46 – 7.24 (m, 6H), 6.24 (dd, *J* = 14.9, 9.9 Hz, 1H), 5.63 (dd, *J* = 9.8, 5.2 Hz, 1H), 2.85 (td, *J* = 15.3, 7.9 Hz, 3H), 2.72 (t, *J* = 7.4 Hz, 1H), 2.65 (t, *J* = 7.3 Hz, 1H), 2.57 – 2.46 (m, 2H), 2.39 (s, 4H), 2.20 – 2.06 (m, 3H), 1.48 (s, 6H). ^{13}C NMR (100 MHz, $CDCl_3$) δ 171.07, 170.98, 170.92, 170.79, 170.53, 170.46, 170.42, 147.65, 147.37, 147.20, 147.15, 147.00, 146.96, 144.93, 136.04, 135.82, 135.77, 133.23, 132.96, 130.70, 130.58, 130.28, 129.65, 128.84, 128.73, 128.50, 127.50, 127.19, 127.16, 125.62, 123.86, 122.49, 121.27, 121.06, 120.98, 116.65, 110.45, 60.53, 35.74, 35.71, 33.51, 33.48, 33.01, 32.97, 27.92, 24.58, 24.52, 24.40, 24.30, 24.20, 23.91, 23.82, 21.94, 21.91, 21.74, 21.69, 21.17, 14.31. Calcd. for $C_{41}H_{40}N_6O_9S_2$ $[M]^+$ 824.2298 found 824.2342.

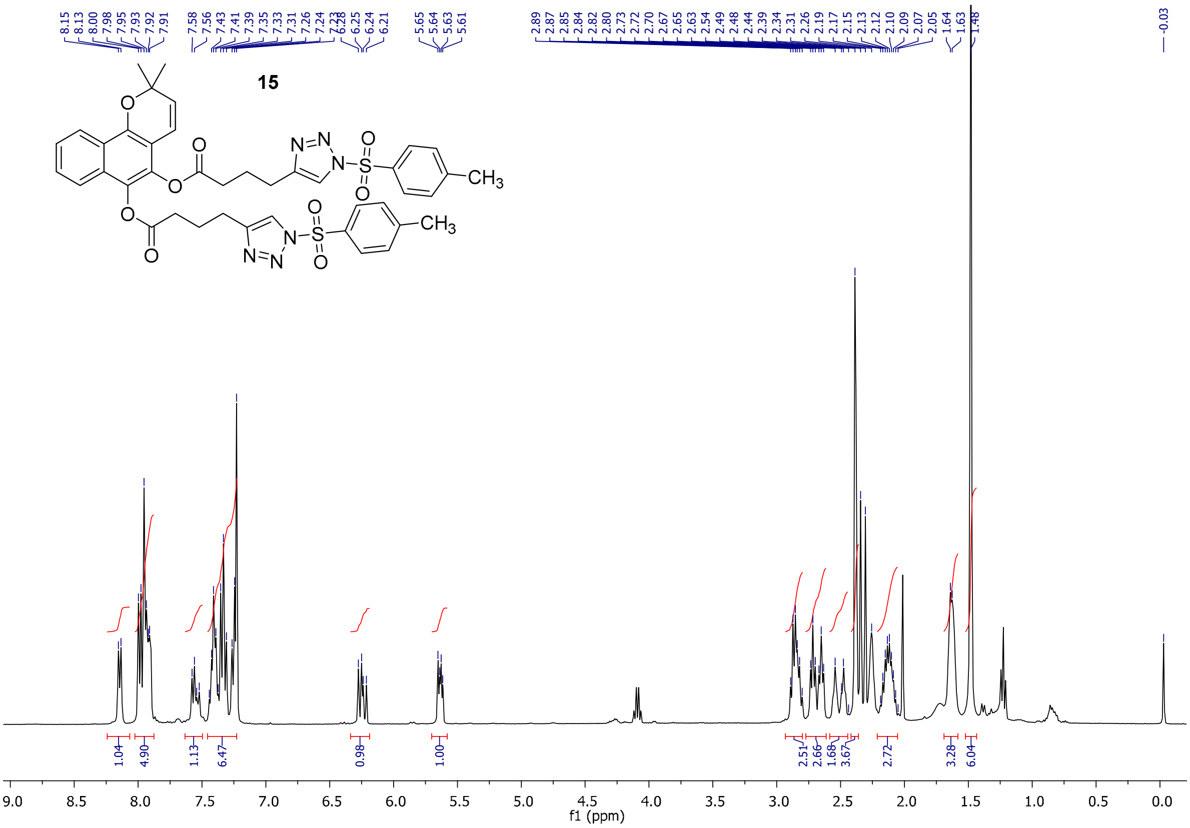

Figure S33. 400 MHz ^{1}H NMR spectra in $CDCl_3$ of **3f**.

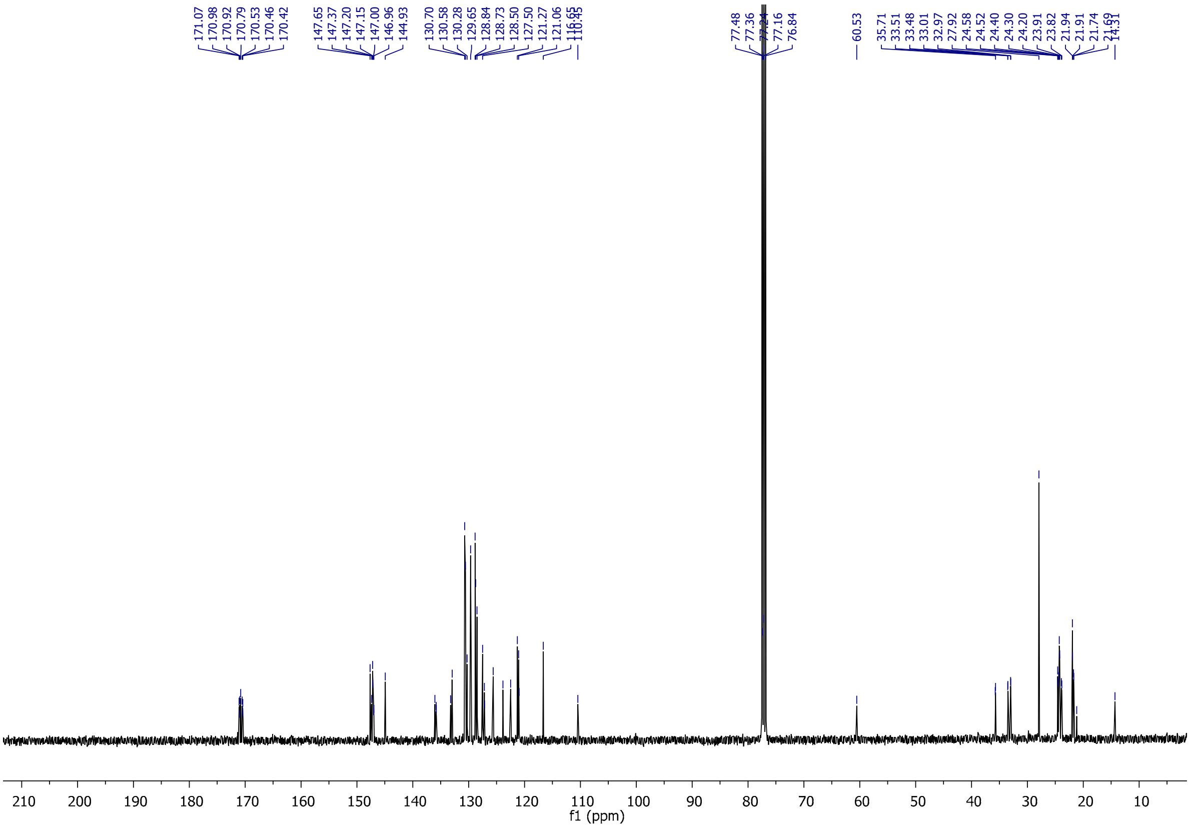

Figure S34. 100 MHz ^{13}C NMR spectra in $CDCl_3$ of **3f**.

Compound 3g. Lapachol (24.2 mg, 0.1 mmol) and 5-hexynoic acid (22 μL, 0.2 mmol), 1-Azido-3-chlorobenzene solution (440 μL, 0.22 mmol, *c* = 0.5 M) were reacted in DCM:THF 1:1 *vv* (2 mL) according to the stepwise esterification-click general procedure. Flash column chromatography purification (*n*-hexane/EtOAc 6:4) afforded **3g** (56 mg, 76%) as a light yellow solid. ^{1}H NMR (400 MHz, $CDCl_3$) δ 8.18 (d, *J* = 7.9 Hz, 1H), 7.85 (s, 2H), 7.80 (dd, *J* = 19.3, 9.3 Hz, 5H), 7.71 – 7.58 (m, 7H), 7.41 (dd, *J* = 15.3, 7.8 Hz, 9H), 6.46 (d, *J* = 10.0 Hz, 1H), 6.32 (d, *J* = 9.9 Hz, 1H), 5.89 (d, *J* = 10.0 Hz, 1H), 5.67 (d, *J* = 9.9 Hz, 1H), 3.83 (dd, *J* = 12.8, 6.4 Hz, 1H), 2.99 (dtd, *J* = 22.8, 14.9, 7.3 Hz, 9H), 2.83 (dd, *J* = 12.9, 6.4 Hz, 4H), 2.76 (t, *J* = 7.2 Hz, 2H), 2.39 – 2.15 (m, 8H), 1.52 (s, 6H), 1.45 (s, 6H), 1.14 (d, *J* = 6.4 Hz, 6H). ^{13}C NMR (100 MHz, $CDCl_3$) δ 171.44, 171.17, 170.98, 170.65, 148.00, 147.25, 138.09, 135.89, 135.65, 134.86, 130.92, 130.66, 130.27, 128.77, 128.70, 127.52, 127.28, 125.62, 124.93, 123.91, 122.54, 121.62, 121.03, 120.78, 120.72, 120.44, 119.50, 118.44, 116.71, 116.63, 115.10, 110.46, 42.35, 33.27, 33.18, 28.28, 27.97, 24.97, 24.92, 24.72, 24.62, 23.61. Calcd. for $C_{39}H_{34}Cl_2N_6O_5$ $[M]^+$ 736.1968 found 736.1942.

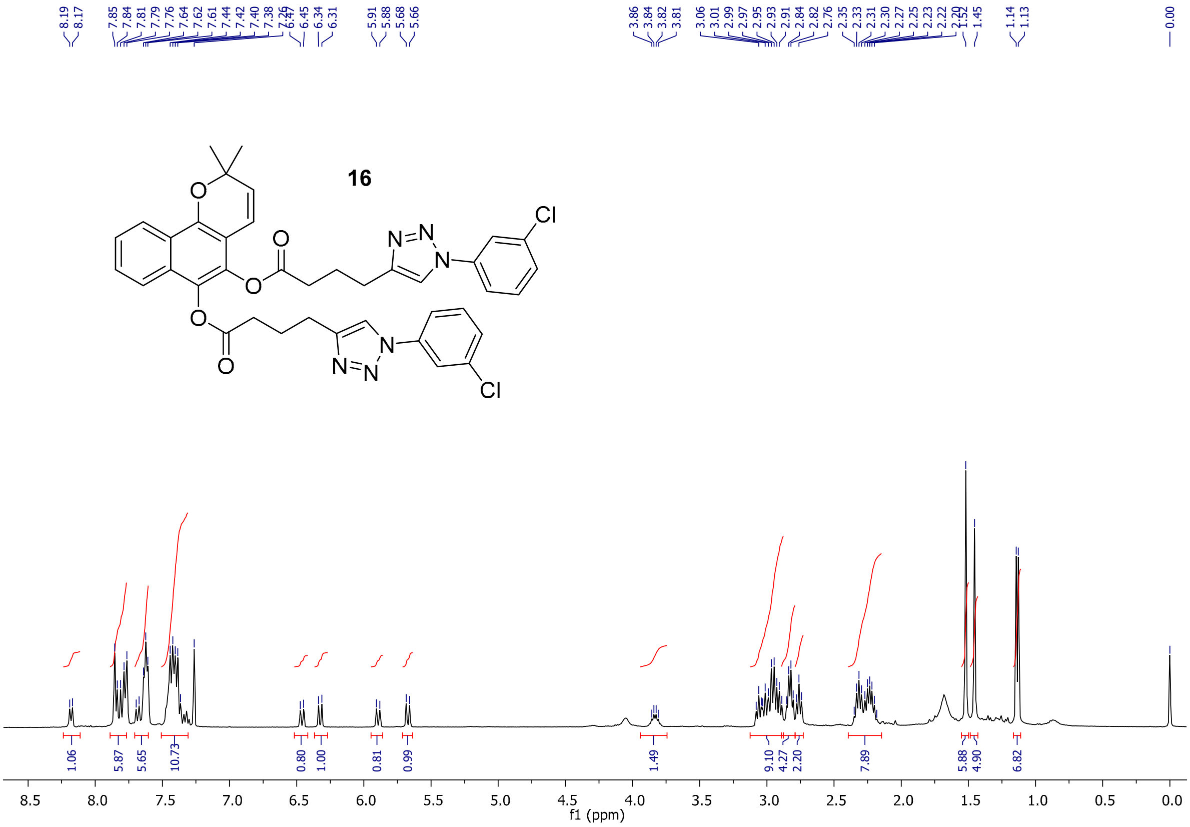

Figure S35. 400 MHz ^1H NMR spectra in $CDCl_3$ of **3g**.

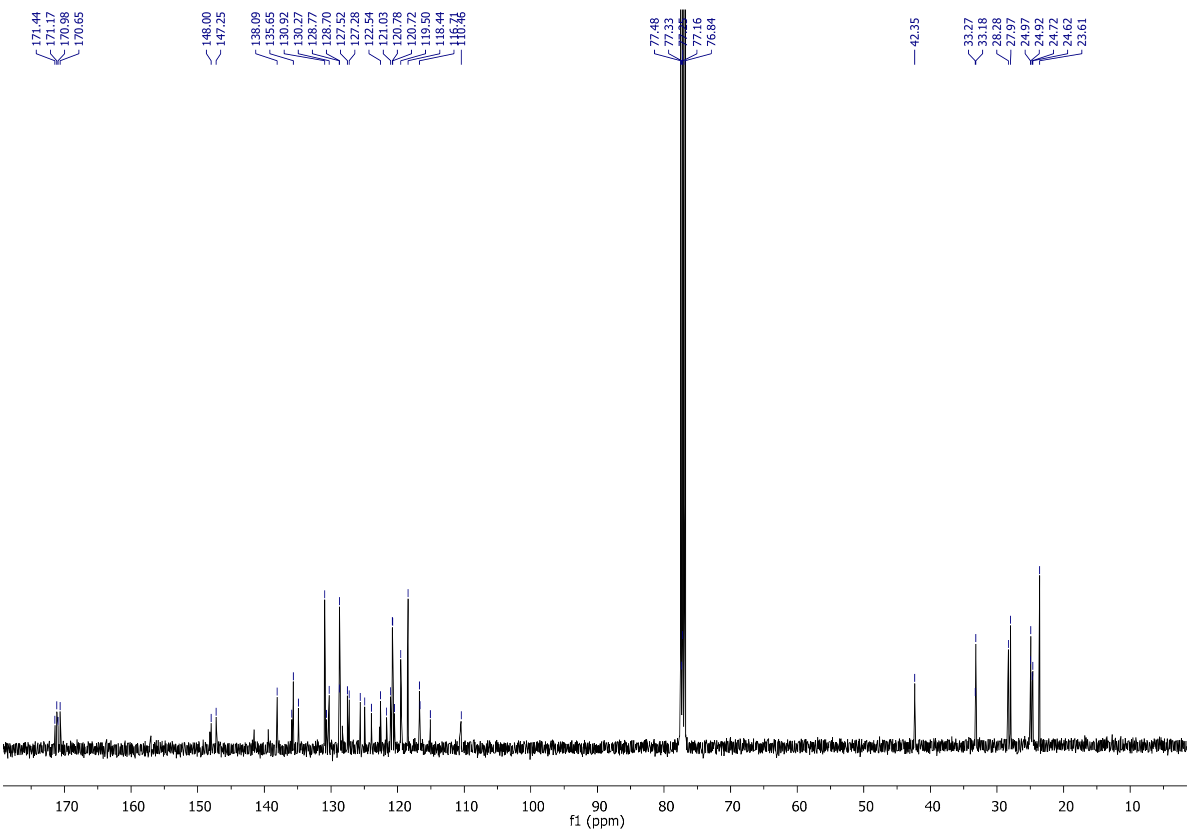

Figure S36. 100 MHz ^{13}C NMR spectra in $CDCl_3$ of **3g**.

Compound 3h. Lapachol (24.2 mg, 0.1 mmol) and 5-hexynoic acid (22 μL, 0.2 mmol), 1-(azidomethyl)-4-nitrobenzene solution (440 μL, 0.22 mmol, *c* = 0.5 M) were reacted in DCM:THF 1:1 *vv* (2 mL) according to the stepwise esterification-click general procedure. Flash column chromatography purification (*n*-hexane/EtOAc 6:4) afforded **3h** (53 mg, 67%) as a light yellow solid. ^{1}H NMR (400 MHz, $CDCl_3$) δ 7.99 (m, 5H), 7.42 (d, *J* = 8.2 Hz, 1H), 7.22 (m, 8H), 6.12 (d, *J* = 9.9 Hz, 1H), 5.50 (d, *J* = 9.9 Hz, 1H), 5.41 (bs, 4H), 2.67 (dd, *J* = 17.2, 7.9 Hz, 4H), 2.59 (t, *J* = 7.3 Hz, 3H), 2.52 (t, *J* = 7.4 Hz, 3H), 2.03 – 1.91 (m, 4H), 1.33 (s, 6H), 1.09 (s, 2H). ^{13}C NMR (100 MHz, $CDCl_3$) δ 171.12, 170.59, 148.06, 147.82, 147.78, 147.19, 142.05, 135.83, 130.60, 130.35, 128.67, 127.52, 127.16, 125.65, 124.31, 123.83, 122.53, 121.59, 120.95, 116.59, 110.41, 53.04, 33.18, 33.15, 29.79, 27.90, 25.03, 24.97, 24.75, 24.66. Calcd. for $C_{41}H_{38}N_8O_9$ $[M]^+$ 786.2762 found 786.2753

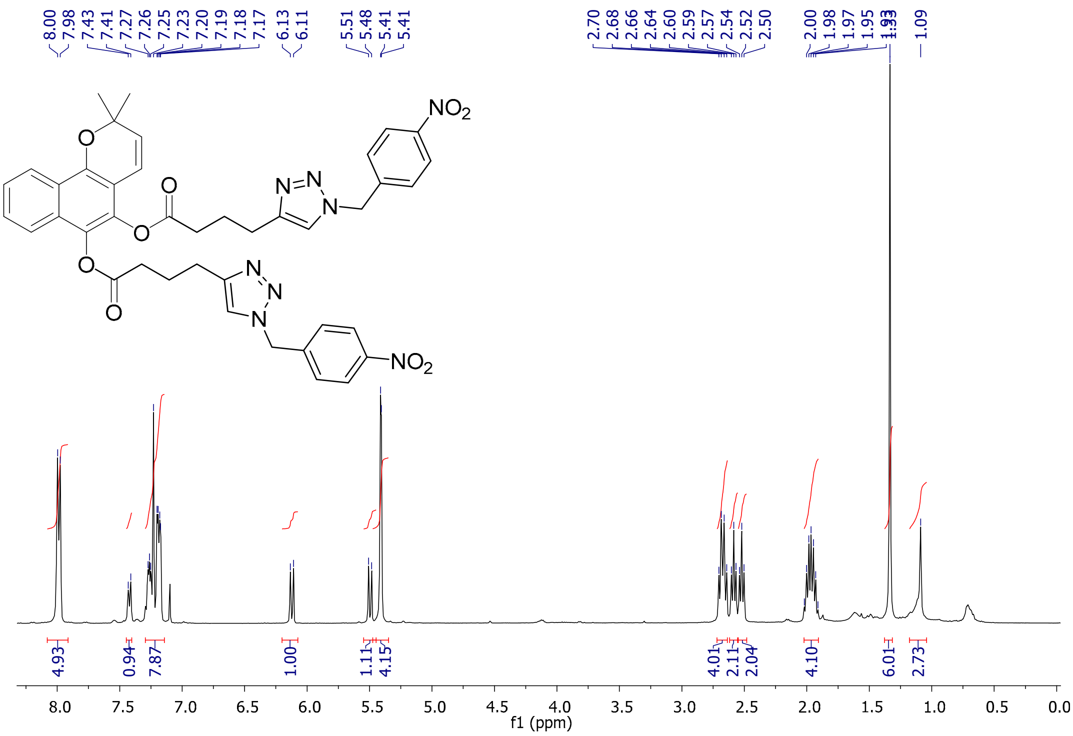

Figure S37. 400 MHz ^{1}H NMR spectra in $CDCl_3$ of **3h**.

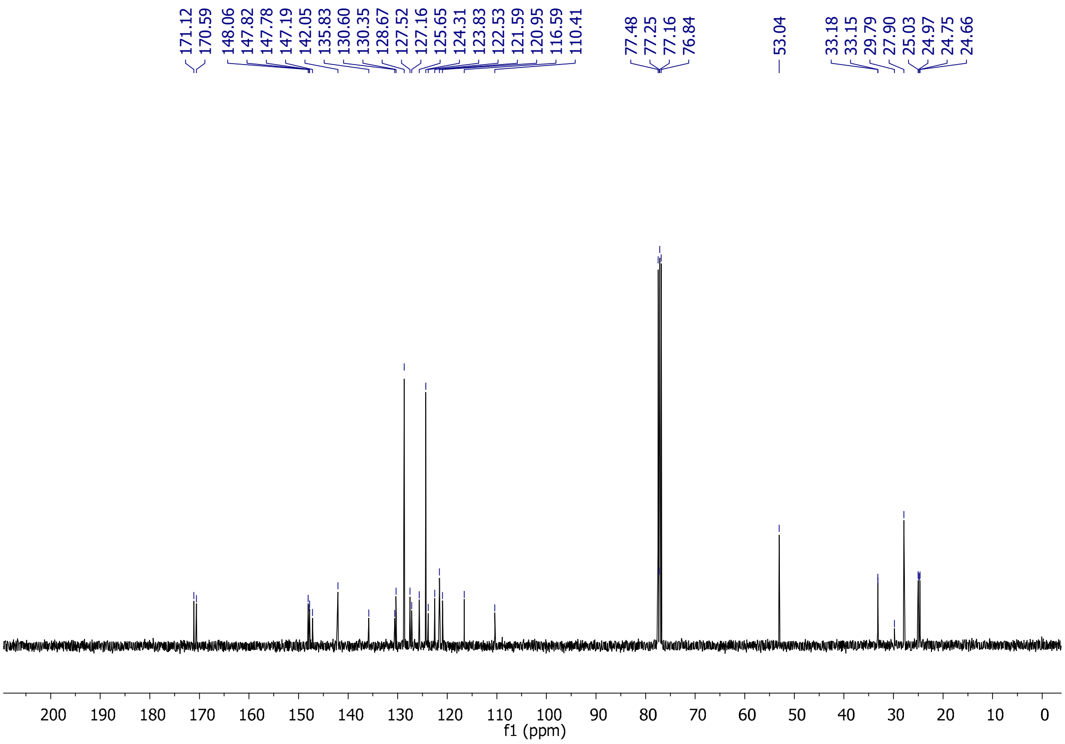

Figure S38. 100 MHz ^{13}C NMR spectra in $CDCl_3$ of **3h**.

Compound 3i. Lapachol (24.2 mg, 0.1 mmol) and 5-hexynoic acid (20 μL, 0.2 mmol), 1-(azidomethyl)-4-bromobenzene solution (440 μL, 0.22 mmol, *c* = 0.5 M) were reacted in

DCM:THF 1:1 *vv* (2 mL) according to the stepwise esterification-click general procedure. Flash column chromatography purification (*n*-hexane/EtOAc 6:4) afforded **3i** (62 mg, 75%) as a light yellow solid. ^{1}H NMR (400 MHz, $CDCl_3$) δ 8.09 (dd, *J* = 6.3, 2.7 Hz, 1H), 7.51 (dd, *J* = 6.2, 2.8 Hz, 1H), 7.42 – 7.29 (m, 5H), 7.23 (s, 2H), 7.04 (d, *J* = 7.6 Hz, 4H), 6.21 (d, *J* = 9.9 Hz, 1H), 5.56 (d, *J* = 9.9 Hz, 1H), 5.35 (s, 4H), 2.74 (dt, *J* = 10.1, 7.5 Hz, 4H), 2.65 (t, *J* = 7.4 Hz, 2H), 2.58 (t, *J* = 7.4 Hz, 2H), 2.11 – 1.98 (m, 4H), 1.43 (s, 6H), 1.17 (s, 2H). ^{13}C NMR (100 MHz, $CDCl_3$) δ 170.53, 170.18, 147.65, 133.72, 132.25, 129.66, 127.39, 125.06, 122.11, 121.07, 116.48, 53.33, 33.06, 27.85, 24.93. Calcd. for $C_{41}H_{38}Br_2N_6O_5$ $[M]^+$ 852.1270 found 852.1296

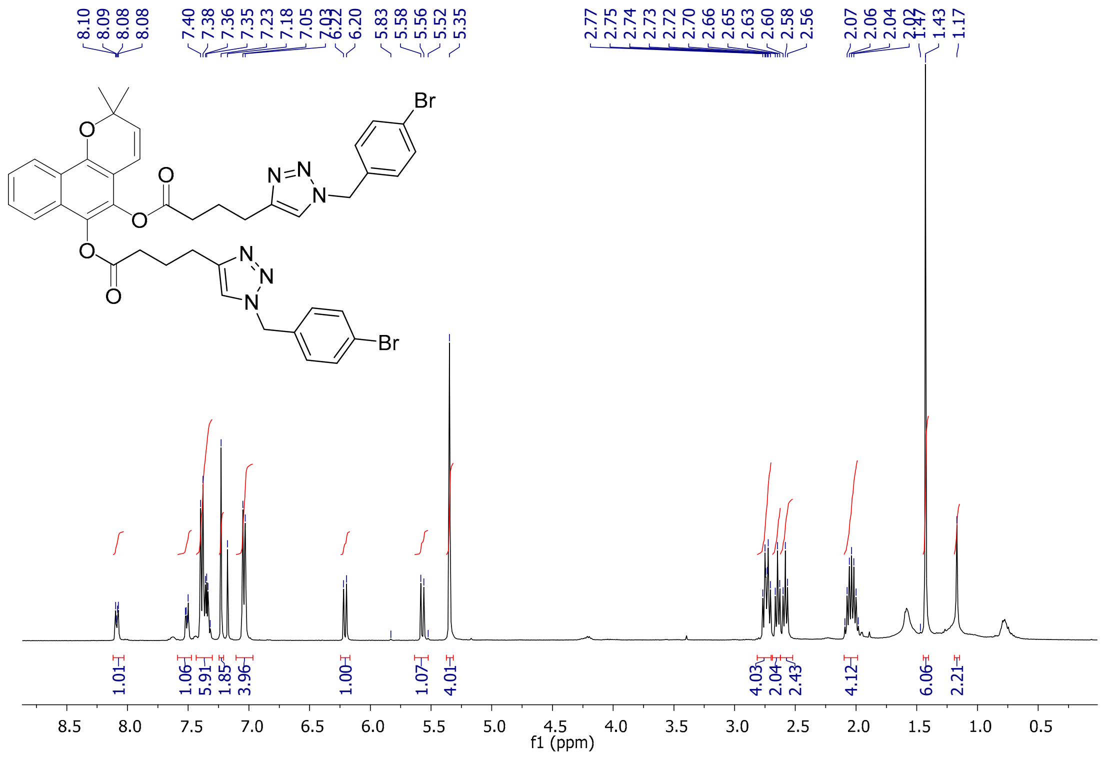

Figure S39. 400 MHz ^{1}H NMR spectra in $CDCl_3$ of **3i**.

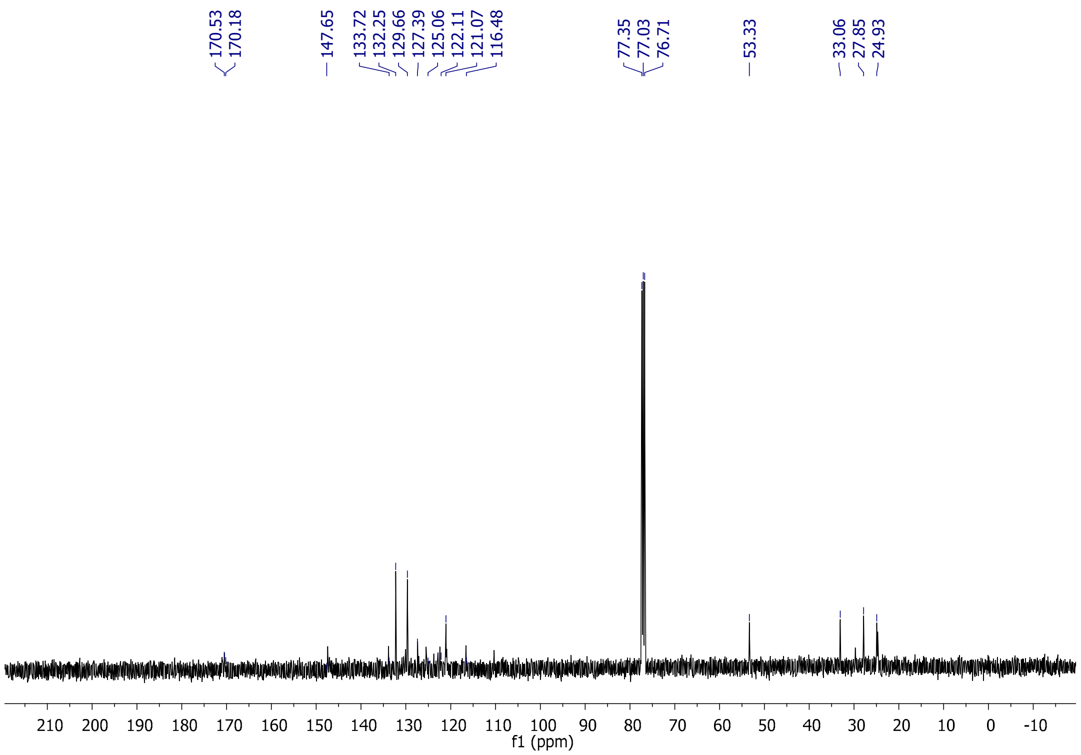

Figure S40. 100 MHz ^{13}C NMR spectra in $CDCl_3$ of **3i**.
